# Glyoxalase 1 sustains the metastatic phenotype of prostate cancer cells via EMT control

**DOI:** 10.1111/jcmm.13581

**Published:** 2018-03-05

**Authors:** Cinzia Antognelli, Rodolfo Cecchetti, Francesca Riuzzi, Matthew J. Peirce, Vincenzo N. Talesa

**Affiliations:** ^1^ Department of Experimental Medicine University of Perugia Perugia Italy

**Keywords:** argpyrimidine, epithelial to mesenchymal transition, glyoxalase 1, hydroimidazolone, metastatic prostate cancer, metformin, miR‐101, TGF‐β1/Smad signalling pathway

## Abstract

Metastasis is the primary cause of death in prostate cancer (PCa) patients. Effective therapeutic intervention in metastatic PCa is undermined by our poor understanding of its molecular aetiology. Defining the mechanisms underlying PCa metastasis may lead to insights into how to decrease morbidity and mortality in this disease. Glyoxalase 1 (Glo1) is the detoxification enzyme of methylglyoxal (MG), a potent precursor of advanced glycation end products (AGEs). Hydroimidazolone (MG‐H1) and argpyrimidine (AP) are AGEs originating from MG‐mediated post‐translational modification of proteins at arginine residues. AP is involved in the control of epithelial to mesenchymal transition (EMT), a crucial determinant of cancer metastasis and invasion, whose regulation mechanisms in malignant cells are still emerging. Here, we uncover a novel mechanism linking Glo1 to the maintenance of the metastatic phenotype of PCa cells by controlling EMT by engaging the tumour suppressor miR‐101, MG‐H1‐AP and TGF‐β1/Smad signalling. Moreover, circulating levels of Glo1, miR‐101, MG‐H1‐AP and TGF‐β1 in patients with metastatic compared with non‐metastatic PCa support our in vitro results, demonstrating their clinical relevance. We suggest that Glo1, together with miR‐101, might be potential therapeutic targets for metastatic PCa, possibly by metformin administration.

## 1 INTRODUCTION

Prostate cancer (PCa) is the most widely diagnosed male cancer in the Western world,^1^, ^2^ and while low‐ and intermediate‐risk PCa patients have a variety of treatment options, metastatic patients are limited to androgen deprivation therapy^3^ which leads, in the majority of patients, to the development of castration‐resistant PCa (CRPC)^4^ and progression to the metastatic stage of the disease (mCRPC) for which treatment options are limited and the prognosis poor. Ongoing research aimed at understanding the molecular bases of metastatic PCa is essential^3^ to identify novel targets for more effective therapies. Glyoxalase 1 (Glo1) is a glutathione‐dependent enzyme that plays a critical cytoprotective role in limiting intracellular accumulation and toxicity of methylglyoxal (MG), a highly reactive dicarbonyl compound mainly formed as a by‐product of glycolysis.^5^ MG readily reacts with lipids, nucleic acids and proteins to form the heterogeneous family of advanced glycation end products (AGEs).^5^ MG‐derived dicarbonyl adducts exert complex pleiotropic effects, including modulation of protein biological activity^6^ and stability,^7^ generation of reactive oxygen species and oxidative stress,^8^, ^9^ which may culminate in distinct biological outcomes.^9^, ^10^, ^11^, ^12^, ^13^, ^14^ Hydroimidazolone (MG‐H1) and argpyrimidine (AP) are major AGEs formed by spontaneous reaction between MG and protein arginine residues.^15^ The levels of MG‐derived AGEs, as well as of AGEs generated by the reaction of the free amino groups of N‐terminal amino acids with carbonyl groups of reducing sugars, have been investigated in different human cancer tissues.^16^, ^17^, ^18^ Results from these studies suggest that the expression pattern of AGEs can be tumour specific^16^ and that their accumulation in cancer tissues may be linked to tumour aggression.^19^ Specifically, in PCa, accumulation of a specific AGE (carboxymethyl lysine, CML) has been recently found in malignant compared with normal tissues where it positively correlates with tumour aggressiveness.^20^ AGEs function as ligand activators for the transmembrane receptor for AGEs (RAGE)^21^ which is overexpressed in a variety of human tumours, including PCa.^20^, ^21^, ^22^, ^23^ Intriguingly, recent data demonstrate that RAGE can regulate Glo1 expression at both mRNA and protein levels.^24^ Glo1 is overexpressed in several human cancers,^5^ where it represents an important tumour survival strategy by preventing accumulation of cytotoxic MG, thereby suppressing MG‐mediated glycation reactions leading to AGE formation. In PCa, we and others have previously shown that Glo1 plays a major role in the progression of this neoplasia.^25^, ^26^ In particular, in highly invasive and metastatic human PC3 PCa cells, Glo1 acts as a pro‐survival factor by eluding apoptosis in a mechanism involving AP and NF‐kB signalling pathway.^27^

An important determinant of metastasis is the epithelial‐to‐mesenchymal transition (EMT), a dynamic transdifferentiation during which cells acquire a migratory and invasive phenotype.^28^ The mechanisms that control the process of EMT in cancer cells are complex and still emerging. In PCa, several key factors contributing to EMT^29^ have recently been identified; transforming growth factor beta (TGF‐β) and miRNAs^30^ have been shown to play a crucial role. In addition, we have recently demonstrated that the Glo1/AP axis can control EMT in a human bronchial model.^11^ The possible role of the Glo1/AP axis in the control of EMT in metastatic PCa has never been investigated. Understanding this circuit in the context of PCa might identify Glo1 as a novel therapeutic target for this lethal stage of the disease. Hence, in this study, we studied whether and how Glo1 might sustain PCa metastatic phenotype as part of the molecular events associated with EMT in DU145 and PC3 human PCa cell lines, models of metastatic PCa. Moreover, to examine this regulatory circuit in a clinically relevant setting, we measured the circulating levels of some of the involved molecules in patients with metastatic compared with non‐metastatic PCa. Finally, we examined the capacity of metformin, a widely used anti‐diabetic drug and an emerging anti‐cancer drug, especially in the field of urologic oncology,^31^, ^32^, ^33^ to inhibit the EMT‐based metastatic phenotype, through control of Glo1 and miR‐101 in both PCa cell lines.

## 2 MATERIALS AND METHODS

### 2.1 Materials

Tissue culture media, foetal bovine serum, penicillin/streptomycin, BCA kit and Laemmli buffer were from ThermoFisher Scientific (Milan, Italy); Roti‐Block was from Roth (Germany). Primary antibodies against Glo1, MMP‐2, MMP‐9, Smad4, lamin β and β‐actin were from DBA (Milan, Italy); primary antibodies against E‐cadherin (E‐cad), zonula occludens‐1 (ZO‐1), vimentin (Vim), N‐cadherin (N‐cad), Snail, TGF‐β1, phospho‐Smad2, phospho‐Smad3 and total Smad2 or Smad3 were from Cell Signalling Technology (Danvers, MA, USA). Anti‐AP mAb was from Antibodies‐online GmbH (Aachen, Germany). The ELISA kit for human TGF‐β1 was purchased from R&D Systems (Milan, Italy). The ELISA kit for MG‐H1 was from DBA (Milan, Italy). Aminoguanidine bicarbonate (AG), a scavenger of free MG^34^ (1 mmol/L, for 3 hours), and metformin (5, 10 and 20 mmol/L for 48 hours) were from Sigma‐Aldrich (Milan, Italy). The TGF‐β type I receptor inhibitor^35^, ^36^ SB431542 (0.5 μmol/L for 1 hour) was from Tocris (Milan, Italy). Control cells for the experiments with agents dissolved in non‐aqueous solvents showed no significant difference with respect to control cells in RPMI‐1640 medium; therefore, all the relative treatments were compared with these latter controls. The biochemical evidence supporting the efficacy of the inhibitors and scavenging agents used in this study was always tested in preliminary experiments, whenever appropriate (data not shown).

### 2.2 Cell lines and cell culture conditions

The human brain metastasis‐derived DU145 and bone metastasis‐derived PC3 cancer cell lines were obtained from the American Type Culture Collection (ATCC) and cultured as per the suppliers' recommendations at 37°C and 5% CO_2_.

### 2.3 Patients

A total of 60 PCa patients were randomly selected from our database from the Urological Department. Patients with bone metastases (n = 30, stage M1) represented the metastatic group, while the non‐metastatic group included 30 patients with stage pT2 (n = 15, cancer confined to the prostate) and stage pT3 (n = 15, extraprostatic extension and/or seminal vesicle involvement). No patients received radiation or hormonal therapy before sample collection. The clinical characteristics of each group are summarized in Table 1. The diagnosis of PCa was confirmed by the histopathological analysis of prostate biopsies with a final Gleason score assigned to every case. Pathological stage categories were determined according to the consolidated 1997 TNM staging system. The research was carried out in accordance with the Declaration of Helsinki and approved by the ethics committee of our hospital. Written consent was obtained from all patients after full explanation of the procedure.

**Table 1:** Clinical characteristics

	Non‐metastatic PCa (n = 30)	Metastatic PCa (n = 30)
Age (y)		
Mean ± SD	66.4 ± 6.3	67.0 ± 7.4
Median	67.0	66.5
Range	55‐77	59‐78
PSA (ng/mL)		
Mean ± SD	11.4 ± 4.6	586.4 ± 254.0
Median	10.0	260.5
Range	2.5‐20	26.4‐2420.0
Gleason score		
Median	6	9
Range	4‐8	8‐10

Non‐metastatic group included: stage pT2 (n = 15, cancer confined to the prostate) and stage pT3 (n = 15, extraprostatic extension and/or seminal vesicle involvement). The metastatic group included patients with bone metastases (stage M1).

PSA, prostate‐specific antigen.

### 2.4 RNA isolation, reverse transcription and qRT‐PCR analysis

RNA isolation, reverse transcription and qRT‐PCR analysis were performed as previously described.^10^, ^27^ Briefly, total cellular RNA was isolated by TRIzol reagent (Life Technologies Italia, Monza, Italy). cDNA was then synthesized from 1 μg of RNA by the RevertAid H Minus First Strand cDNA Synthesis Kit (Life Technologies Italia, Monza, Italy). The expression of target genes vs β‐actin was evaluated by qRT‐PCR on an MX3000P real‐time PCR system (Agilent Technology, Milan, Italy). Primers pairs and TaqMan probes for PCR are as follows: Glo1 5′‐CTCTCCAGAAAAGCTACACTTTGAG‐3′ (sense, 400 nmol/L) and 5′‐CGAGGGTCTGAATTGCCATTG‐3′ (antisense, 400 nmol/L), 5′‐FAM‐TGGGTCGCATCATCTTCAGTGCCC‐TAMRA‐3′ (TaqMan probe, 200 nmol/L); β‐actin 5′‐CACTCTTCCAGCCTTCCTTCC‐3′(sense, 600) and 5′‐ACAGCACTGTGTTGGCGTAC‐3′ (antisense, 600 nmol/L), 5′‐TEXASRED‐TGCGGATGTCCACGTCACACTTCA‐BHQ‐3′ (TaqMan probe, 200 nmol/L); PAI1 5′‐ACATGTTTAGTGCAACCCTG‐3′ (sense, 400 nmol/L) and 5′‐GGTCTATAACCATCTCCGTG‐3′ (antisense, 400 nmol/L); ZEB1 5′‐TCAAGTACAAACACCACCTG‐3′ (sense, 400 nmol/L) and 5′‐TGGCGAGGAACACTGAGA‐3′ (antisense, 400 nmol/L)^37^; HMGA2 5′‐CCCAAAGGCAGCAAAAACAA ‐3′ (sense, 400 nmol/L) and 5′‐GCCTCTTGGCCGTTTTTCTC ‐3′(antisense, 400 nmol/L).^38^ PCR for PAI1, ZEB1 and HMGA2 was performed by SYBR Green biochemistry. For normalizing purposes, β‐actin detection was performed by the same biochemistry with the primers above described. PCR was performed in a total volume of 25 μL, containing 250 ng of cDNA, 1X Brilliant or SYBR Green QPCR Master Mix (Agilent Technology, Milan, Italy), ROX reference dye and the specific primers/probes. The thermal cycling conditions were as follows: 1 cycle at 95°C for 10 minutes, followed by 45 cycles at 95°C for 20 seconds, and 55°C or 60°C for 1 minute. Data for comparative analysis of gene expression were obtained by the 2^−ΔΔCt^ method.^39^

### 2.5 Cell lysate preparation

Total protein extraction was performed by lysing the cells with pre‐cooled radio‐immunoprecipitation assay (RIPA) lysis buffer. The nuclear extracts were prepared with the FractionPREP Cell Fractionation Kit (BioVision, Florence, Italy), according to the manufacturer's instructions. Protein concentration was determined with a bicinchoninic acid (BCA) kit (Pierce), with bovine serum albumin as a standard.

### 2.6 SDS‐PAGE and Western blot analysis

SDS‐PAGE and Western blot analysis were performed as previously described.^11^, ^40^ Briefly, samples of equal protein concentration (20‐40 μg) were treated with Laemmli buffer, boiled for 5 minutes, resolved on 10%, 12% or 15% SDS‐PAGE and then blotted onto a nitrocellulose membrane by the iBlot Dry Blotting System (Invitrogen, Milan, Italy). Non‐specific binding sites were blocked in Roti‐Block for 1 hour at room temperature and then incubated overnight at 4°C with an appropriate dilution of the primary specific Abs. After being washed with Tris‐buffered saline/Tween, antigen‐antibody complexes were detected by incubation of the membranes for 1 hour at room temperature with the appropriate horseradish peroxidase‐conjugated secondary Ab and visualized by the ECL system (Microtech, Naples, Italy). As internal loading controls, all membranes were subsequently stripped off the first Ab in a stripping buffer (100 mmol/L 2‐mercaptoethanol, 2% SDS and 62.5 mmol/L Tris‐HCl, pH 6.8) and reprobed with the appropriate housekeeping Ab.

### 2.7 Glyoxalase 1 enzyme activity

The activity of Glo1 was assessed as previously described in lysates either from cell lines^40^ or from red blood cells.^41^, ^42^, ^43^ Glo1 enzymatic activity was assayed by an established method.^44^ Briefly, the assay solution contained 0.1 mol/L sodium phosphate buffer, pH 7.2, 2 mmol/L MG and 1 mmol/L GSH. The reaction was monitored spectrophotometrically by following the increase in absorbance at 240 nm and 25°C. One unit of activity is defined as 1 μmol of S‐D‐lactoylglutathione produced per minute.

### 2.8 siRNA transfection

**Figure 1:**
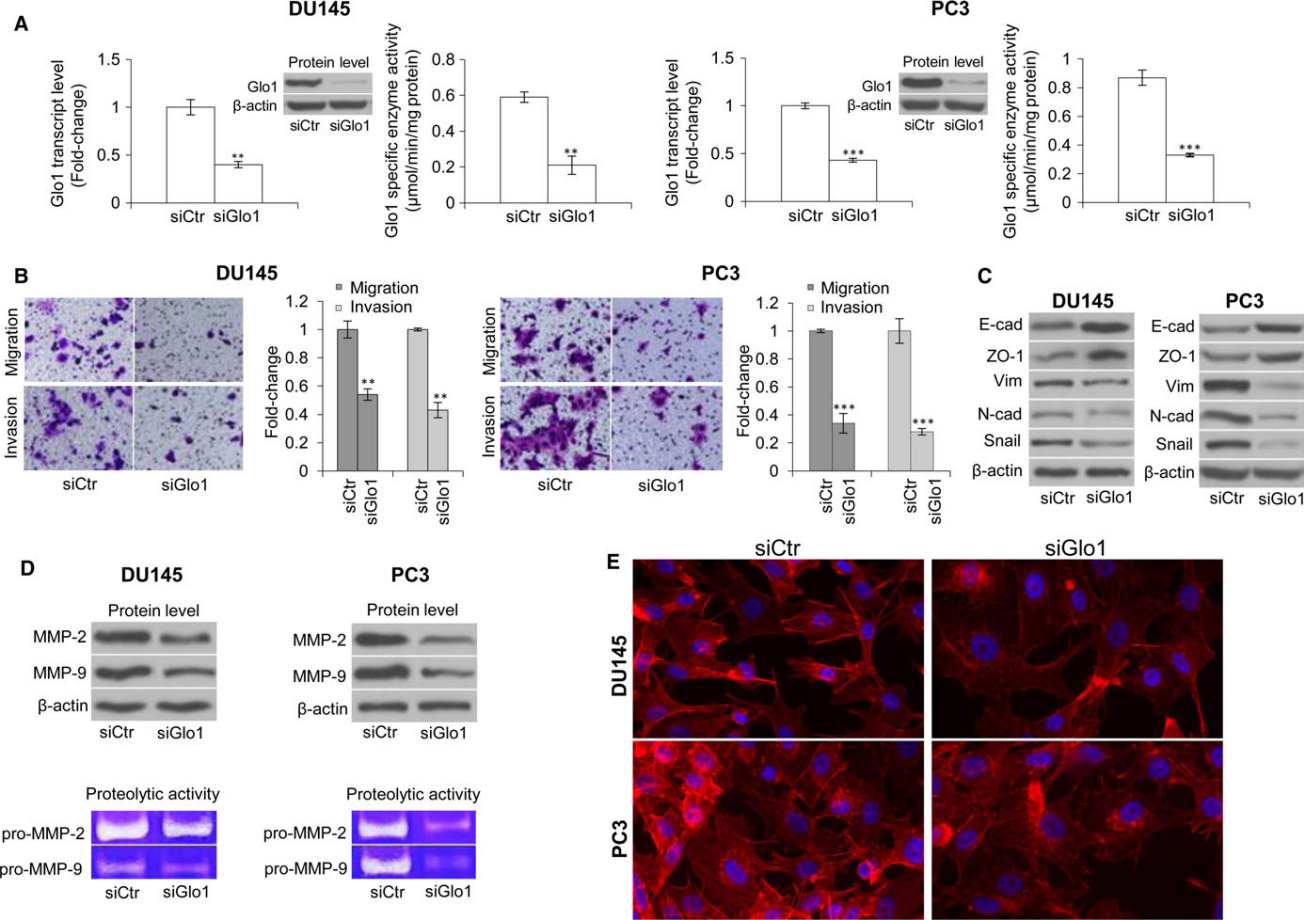
Glyoxalase 1 (Glo1) sustains migration and invasion activities of metastatic DU145 and PC3 cell lines via epithelial‐to‐mesenchymal transition (EMT) control. (A) Transient knock‐down of Glo1 (siGlo1) in DU145 and PC3 cells was confirmed by evaluating Glo1 transcript level, by qRT‐PCR, protein level, by Western blotting, and specific enzyme activity, by spectrophotometric assay. Glo1 silencing in DU145 and PC3 cells significantly affected migration and invasion capabilities, evaluated by specific assays (B), EMT, evaluated by Western blot analysis of the epithelial markers E‐cadherin (E‐cad) and zonula occludens‐1 (ZO‐1), of the mesenchymal markers vimentin (Vim) and N‐cadherin (N‐cad) and the transcriptional factor Snail (C), MMP‐2 and MMP‐9 expression (top panel), evaluated by Western blotting, or MMP‐2 and MMP‐9 activity (bottom panel), evaluated by the gelatin zymographic method (D), and cytoskeleton organization, evaluated by immunofluorescence staining of F‐actin (E). The Western blots were obtained by the appropriate Abs. The blots were stripped off the bound Abs and reprobed with anti‐β‐actin to confirm equal loading. The Western blots shown are representative of three independent experiments. The histograms indicate mean ± SD of three different cultures, and each was tested in triplicate. Distance scale is 10 μm. siCtr: control (non‐specific siRNA). **P < .01, ***P < .001 vs siCtr cells

Prostate cancer cells were transiently transfected with a pool of four small interfering RNA (siRNA) oligonucleotides targeting Glo1(siGlo1) or Glo2 (siGlo2) (ON‐TARGET plus SMART pool siRNA) or with a pool of non‐targeting siRNA oligonucleotides (siCtr) (ON‐TARGET plus siCONTROL) as a negative control to exclude off‐targets (all from Dharmacon RNA Technologies, Carlo Erba, Milan, Italy), with DharmaFECT 2 transfection reagent (Dharmacon RNA Technologies, Carlo Erba, Milan, Italy), according to the manufacturer's instructions and as previously described.^27^, ^45^ The biochemical (protein level and enzymatic activity) and molecular (transcript level) evidence of Glo1 gene silencing was verified by Western blotting and spectrophotometric assay, or qRT‐PCR, respectively (Figure 1A). The same experimental design was used for glyoxalase 2 (Glo2) gene silencing (data not shown). Mock transfection was performed to control potential effects due to the transfection reagent. As no significant differences were found between siCtr‐ or mock‐treated and non‐transfected cells on the biological phenomena under investigation (Figure S1) in either DU145 or PC3 cells, the observed changes were shown with respect only to siCtr‐exposed cells.

### 2.9 Ectopic expression of Glo1 and Glo2

Cells were infected with a transfection‐ready plasmid‐encoding Glo1 (pCMV‐Glo1) or Glo2 (pCMV‐Glo2) or control DNA plasmid (pCMV‐Ctr), according to the manufacturer's instructions (OriGene, Tema Ricerca, Bologna, Italy).

### 2.10 Transwell migration and invasion assays

Transwell migration and invasion assays were carried out as described by Malkoski et al.^46^ Briefly, transwell assays were conducted with 200 μL Boyden's blindwell chambers (50 mm^2^ area) and polyvinyl pyrrolidone‐free polycarbonate membranes with 12‐μm pores (Neuro Probe, Warwick, UK^47^). Migration membranes were coated with 0.01% porcine gelatin, and invasion membranes were coated with 5% growth factor‐reduced Matrigel (BD Biosciences, Milan, Italy). Non‐migrating cells were removed, and membranes were fixed, stained and photographed at 100×.

### 2.11 Gelatin zymography

Gelatin zymography was performed according to Shin et al.^48^ Briefly, the protein lysates were denatured by mixing 5× gel‐loading buffer containing 0.1 mol/L Tris‐HCl, pH 6.8, 50% glycerol, 2% SDS and 0.1% bromophenol and electrophoretically separated on a polyacrylamide gel containing 0.2% gelatin. The resolved proteins in the gel were washed and renatured by the exchange of SDS with non‐ionic detergent Triton X‐100 contained in washing buffer (50 mmol/L Tris‐HCl at pH 7.5, 150 mmol/L NaCl, 10 mmol/L CaCl_2_, 0.02% NaN_3_ and 1 μmol/L ZnCl_2_) for 24 hours at 37°C with shaking. The incubated gel was stained with Coomassie Blue R‐250, and the proteolytic activities of the MMPs were detected against a blue background as clear bands that resulted from degradation of gelatin.

### 2.12 Immunofluorescence microscopy

Immunofluorescence for F‐actin detection was performed as previously described.^11^, ^49^ Briefly, the cells grown on a coverslip were fixed in 3.7% paraformaldehyde, permeabilized in 0.1% Triton X‐100 and treated with phalloidin‐tetramethylrhodamine B isothiocyanate (TRITC) mAb. The cells were nuclear‐stained with 4′,6‐diamidino‐2‐phenylindole (DAPI). The coverslips were then mounted on slides with PBS/glycerol (1:1), and images were obtained by fluorescence light microscopy (Zeiss, Germany).

### 2.13 miRNA isolation, analysis and transfection

miRNA isolation was performed according to the protocol of the High Pure miRNA Isolation Kit (Roche, Milan, Italy). For miRNA analysis, reverse transcription and PCR were carried out with the Bulge‐Loop miRNA qRT‐PCR primer set (RiboBio, Guangzhou, China).^50^ The miRNA expression relative to U6 (RiboBio) was determining with 2^−ΔΔCt^ method.^39^ miR‐101 inhibition was performed according to Dai et al.^50^ Briefly, 100 nmol/L scrambled 22‐nt control (miRNA‐Scr, with no homology to the mammalian genome) or 150 nmol/L miR‐101 inhibitor (designed and synthesized by RiboBio) was mixed with Lipofectamine 2000 and then left at room temperature for 20 minutes. Before the mixture was added, 1 mL fresh medium was added to each well, and then, the mixture was added for 12‐hours incubation. miR‐101 overexpression was performed by pCMV‐miR‐101 overexpression (OriGene, Rockville, MD) and control (pCM‐Ctr) plasmids transfected with HiPerFect Transfection Reagent (Qiagen‐Italy, Milan, Italy), according to the manufacturer's instructions. As no significant differences were found between pCM‐Ctr‐treated (or miR‐Scr‐) and untreated cells, comparisons were shown with respect to pCM‐Ctr (or miR‐Scr). In cotransfection experiments, miR‐101 inhibitor was added 24 hours after pCMV‐miR‐101 overexpression and left for additional 12 hours. After that, results were evaluated. Plasma miR‐101 levels were detected according to Zheng et al.^51^ Circulating miR‐101 was detected according to Cheng et al.^52^

### 2.14 Affinity purification of MG‐modified proteins and amino acid sequence analysis

Affinity purification of MG‐modified proteins and amino acid sequence analysis was performed according to Sakamoto et al.^53^ Briefly, DU145 and PC3 cells (10^9^ cells) were lysed in buffer A (50 mmol/L Hepes‐KOH, pH 7.0, 0.1% CHAPS, 2 mmol/L EDTA, 10 mmol/L dithiothreitol, 10% glycerol) by Dounce homogenization. The lysate was clarified through successive centrifugation steps (a 1000 × *g* spin followed by a 10 000 × *g* spin) at 4°C. Mouse monoclonal anti‐argpyrimidine antibody (Antibodies‐online, GmbH, Aachen, Germany) was coupled with Affi Gel‐HZ (BioRad), according to the supplier's instructions. The cell lysate was applied on an immunoaffinity column equilibrated with buffer A. The column was washed with buffer A containing 0.5 mol/L NaCl to remove unbound proteins. Bound proteins were eluted with 0.2 mol/L glycine‐HCl (pH 2.5), resolved on SDS‐PAGE and visualized by Coomassie Blue staining. Purified 40‐kD protein was digested with lysyl endopeptidase (Wako Chemicals GmbH, Germany) in 50 mmol/L Tris‐HCl, pH 8.5, at 37°C for 16 hours. The peptide fragments obtained were separated by reversed‐phase HPLC. Elution profiles were monitored by absorbance at 220 nm, and the peptide fragments were manually collected. The amino acid sequence of the peptide fragments fractionated by HPLC was defined with a PPSQ‐21 gas‐phase sequencer (Shimadzu, Kyoto, Japan).

### 2.15 Immunoprecipitation

Cells were lysed in ice‐cold lysis buffer (PBS, 1% Triton X‐100, 12 mmol/L sodium deoxycholate and 0.1% SDS) with a cocktail of proteinase inhibitors. The lysate was incubated with protein G‐agarose conjugated with mouse anti‐TGF‐β mAb (TGF‐beta, TGFb antibody, Antibodies‐online, GmbH, Aachen, Germany) and mouse anti‐Hsp40 mAb (HSP40/Hdj1 mAb, 2E1, Enzo Life Science, 3V Chimica S.r.l., Rome, Italy), and Western blot analysis was performed as described above, with an anti‐AP Ab.

### 2.16 Plasmid constructs and luciferase reporter assay

To construct the Glo1 expression plasmids, the wild‐type or mutant 3′ UTR of Glo1 gene was cloned into the pEZX vector. All clones were verified by DNA sequencing (GeneCopoeia, Fulengen, China). For the luciferase reporter assay, DU145 and PC3 cells were seeded into 24‐well plates and incubated overnight. Cells were then cotransfected with plasmid containing pEZX/Glo1‐3′‐UTR or pEZX/Glo1‐3′‐UTR‐mutant and miR‐101 or miR‐Ctrl (GeneCopoeia, Fulengen, China), with Lipofectamine 2000 (Invitrogen, Milan, Italy). After 24 hours of transfection, the cells were lysed and Firefly and Renilla luciferase activities were measured by a microplate reader by the Luc‐Pair miR Luciferase Assay (GeneCopoeia, Fulengen, China). Normalized data were obtained by calculating the ratios of Firefly to Renilla luciferase luminescence. All experiments were performed in triplicate.

### 2.17 TGF‐β1 detection

TGF‐β1 concentration in cell culture supernatants and plasma was measured by ELISA, according to the manufacturer's instructions.

### 2.18 Measurement of circulating MG‐H1 and AP levels

AP measurement in plasma was performed according to Raj et al.^54^ OxiSelect™ Methylglyoxal Competitive ELISA Kit was used to detect MG‐H1 levels.^55^

### 2.19 Statistical analysis

Analysis of data was performed by SPSS 11.0 for Windows. The data were statistically analysed by either Student's *t* test or ANOVA followed by the Bonferroni correction for multiple comparisons, when appropriate. Correlation analyses were carried out with the Spearman's correlation test. *P* values of less than .05 were considered statistically significant.

## 3 RESULTS

### 3.1 Glo1 sustains migration and invasion in metastatic DU145 and PC3 cells via EMT control

To investigate the possible involvement of Glo1 in the metastatic phenotype of PCa cell lines, we transiently knocked down Glo1 in DU145 and PC3 PCa cells and evaluated cell migration and invasion, phenomena typically associated with metastasis.^56^ Following Glo1 silencing (siGlo1 transfection) (Figure 1A), DU145 and PC3 cells exhibited significantly reduced migration and invasion abilities compared with control (siCtr‐transfected) cells (Figure 1B), suggesting that Glo1 sustained migration and invasion of these metastatic PCa cells. In PCa cells, EMT is considered a major prerequisite for acquiring migratory/invasive phenotype and subsequent metastasis.^29^, ^57^ Therefore, we studied whether Glo1 could sustain DU145 and PC3 metastatic phenotype via EMT control. To this end, EMT was studied by analysing the expression of E‐cad and ZO‐1, markers of epithelial cells, vimentin and N‐cad, markers of mesenchymal cells, Snail, a crucial transcription factor involved in EMT of PCa cells^58^ and MMP‐2 and MMP‐9, whose mRNA and protein are found at increased levels in the serum and tissue samples from PCa patients and are correlated with metastatic disease.^59^ As shown in Figure 1C, Glo1 silenced (siGlo1) DU145 and PC3 cells were characterized by increased expression of E‐cad and ZO‐1 and concurrently decreased expression of vimentin, N‐cad and Snail compared with siCtr cells. Moreover, Glo1 silencing reduced MMP‐2 and MMP‐9 protein expression and activity, while these remained unaffected in siCtr‐treated DU145 or PC3 cells (Figure 1D). Finally, Glo1 silencing also resulted in the reorganization of the cytoskeleton (Figure 1E). In particular, immunofluorescence staining of F‐actin showed a clear decrease in staining intensity and reduction of actin stress fibres in siGlo1‐treated compared with siCtr‐treated DU145 and PC3 cells (Figure 1E). Collectively, our findings showed that Glo1 sustained the metastatic phenotype of the human DU145 and PC3 PCa cell lines by EMT control.

### 3.2 Glo1‐dependent control of EMT occurs via hydroimidazolone (MG‐H1) and AP depletion in DU145 and PC3 cells

**Figure 2:**
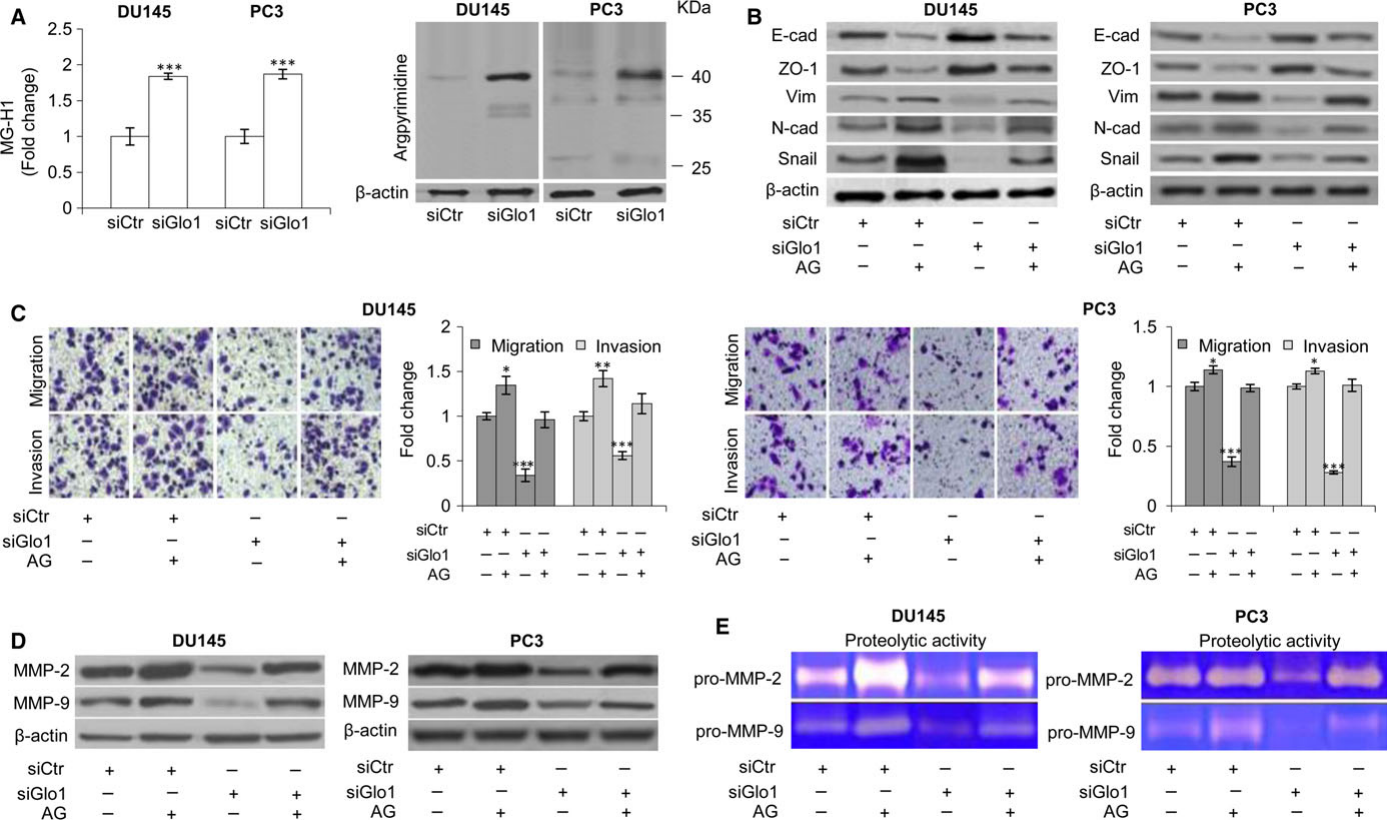
Glyoxalase 1 **(**Glo1)‐dependent positive control of epithelial‐to‐mesenchymal transition (EMT) occurs via hydroimidazolone (MG‐H1) and argpyrimidine (AP) depletion in DU145 and PC3 cells. (A) Glo1 silencing (siGlo1) in DU145 and PC3 cells significantly affected the intracellular levels of both MG‐H1, measured by a specific ELISA kit, and AP, evaluated by Western blot. Pre‐treatment with aminoguanidine (AG) proved MG‐derived AGEs role in controlling (B) EMT, evaluated by Western blot analysis of the epithelial markers E‐cadherin (E‐cad) and zonula occludens‐1 (ZO‐1) or mesenchymal markers vimentin (Vim), N‐cadherin (N‐cad) and Snail, (C) migration and invasion capabilities, evaluated by specific assays, and MMP‐2 and MMP‐9 expression, evaluated by Western blotting (D), or activity (E), evaluated by the gelatin zymographic method. The Western blots were generated by the appropriate Abs. The blots were stripped off the bound Abs and reprobed with anti‐β‐actin to confirm equal loading. AP detection yielded multiple bands. Here, only the most intense band was shown. The Western blots shown are representative of three independent experiments. The histograms indicate mean ± SD of three different cultures, and each was tested in triplicate. siCtr: control (non‐specific siRNA). (−) untreated and (+) treated cells. *P < .05, ***P < .001 vs siCtr cells

To investigate a possible mechanism by which Glo1 sustained the metastatic phenotype of DU145 and PC3 cells via EMT control, we focused on MG‐H1 and AP, AGEs generated from the spontaneous modification of arginine residues^15^ by MG, the substrate of Glo1. Besides, AP, like other AGEs, has been reported to be involved in EMT control in a context‐dependent manner.^11^, ^60^ With an ELISA kit specific to MG‐H1 and an antibody specific to AP‐modified residues to use in Western blotting, we detected the intracellular levels of these two adducts. As illustrated in Figure 2A, compared with siCtr cells, siGlo1 treatment induced MG‐H1 and AP accumulation in both DU145 and PC3 cells. In particular, as to AP immunodetection, we observed a major band with an approximate molecular weight of 40 kD whose intensity was markedly increased by siGlo1 treatment. To further test the hypothesis that MG‐H1 and AP play a key role in the phenotypic changes associated with siRNA‐mediated Glo1 depletion, we next examined the effect of AG, a scavenger of free MG,^61^ on various aspects of the EMT phenotype lost upon siGlo treatment. Thus, as depicted in Figure 2, upon Glo1 silencing, AG exposure was able to restore the EMT‐associated phenotype, at the level of expression of epithelial cell markers (Figure 2B), migration and invasion (Figure 2C) as well as MMP‐2 and MMP‐9 expression (Figure 2D) and activity (Figure 2E) with respect to siCtr cells. Together, these findings support a mechanism whereby Glo1 acts by suppressing intracellular levels of MG‐H1 and AP, to sustain EMT and the metastatic phenotype of DU145 and PC3 cells.

### 3.3 Glo1‐dependent MG‐H1 and AP depletion triggers EMT via TGF‐β1/Smad signalling pathway in DU145 and PC3 cells

**Figure 3:**
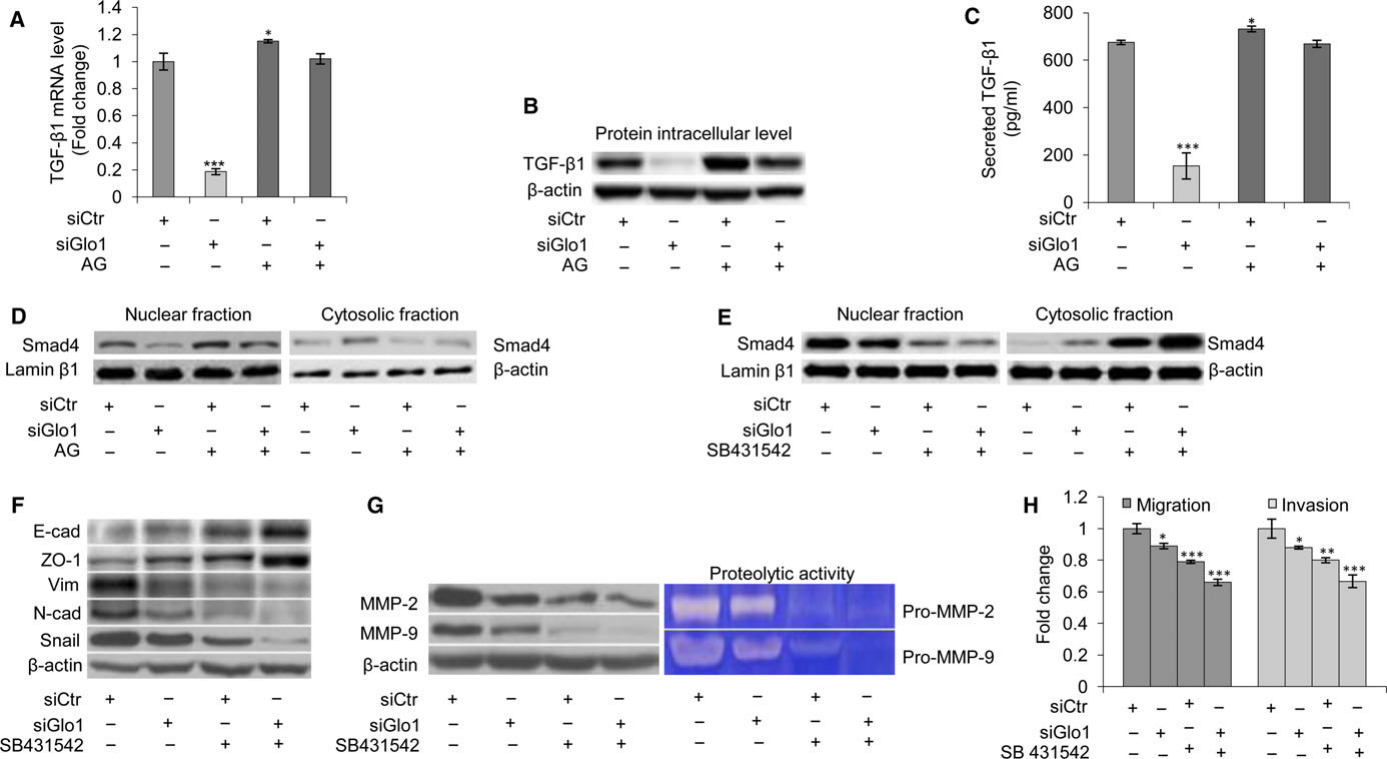
Glyoxalase 1 (Glo1)‐dependent MG‐H1 and argpyrimidine (AP) depletion triggers epithelial‐to‐mesenchymal transition (EMT) via TGF‐β1/SMAD4 signalling pathway in PC3 cells. (A) Glo1 silencing (siGlo1) in PC3 cells significantly affected TGF‐β1 mRNA levels, evaluated by qRT‐PCR, (B) TGF‐β1 protein intracellular levels, evaluated by Western blot, and (C) TGF‐β1 secreted levels, evaluated by a specific ELISA kit. Pre‐treatment with aminoguanidine (AG) proved Glo1‐dependent Hsp40‐modified AP‐mediated role in controlling TGF‐β1 expression (A, B, C). (D) Glo1 silencing (siGlo1) and AG pre‐treatment significantly affected Smad4 activation, evaluated both in nuclear and in cytoplasmic fractions of PC3 cells by Western blot, proving Glo1‐dependent Hsp40‐modified AP‐mediated role in controlling TGF‐β1/Smad4 signalling pathway. Inhibition of TGF‐β1 signalling by SB431542 treatment demonstrated TGF‐β1 role in controlling (E) Smad4 activation, evaluated at nuclear and cytoplasmic levels by Western blot, (F) EMT, evaluated by Western blot analysis of the epithelial markers E‐cadherin (E‐cad) and zonula occludens‐1 (ZO‐1) or mesenchymal markers vimentin (Vim), N‐cadherin (N‐cad) and Snail, (G) MMP‐2 and MMP‐9 expression, evaluated by Western blotting or activity, evaluated by the gelatin zymography, and (H) migration and invasion capabilities, evaluated by specific assays. The Western blots were obtained by the appropriate Abs. The blots were stripped off the bound Abs and reprobed with anti‐β‐actin or lamin β1 to confirm equal loading. The Western blots shown are representative of three independent experiments. The histograms indicate mean ± SD of three different cultures, and each was tested in triplicate. siCtr: control (non‐specific siRNA). (−) untreated and (+) treated cells. *P < .05, **P < .01, ***P < .001 vs siCtr cells

The TGF‐β1 signalling pathway has been linked to the invasion and metastasis of PCa cells via EMT induction.^62^ Signalling via TGFβ1 is mediated by the Smad‐dependent signalling pathway. Upon binding of TGF‐β1 to its receptor, receptor‐regulated Smad2/3 proteins become phosphorylated and associate with Smad4. This complex translocates to the nucleus, binds to DNA and regulates transcription of specific genes, such as Snail, a key transcription factor that promotes migration and invasion of cancer cells, including PCa.^63^ To address the possible role of the TGF‐β1 signalling pathway in the induction of EMT by Glo1‐dependent MG‐H1 and AP depletion, we studied the nuclear translocation of Smad4, the central mediator of TGF‐β1‐induced EMT.^64^ Thus, in PC3 cells, we found that upon siGlo1 silencing, mRNA (Figure 3A), protein expression (Figure 3B) or secretion (Figure 3C) of TGF‐β1 and Smad4 activation (Figure 3D) were all significantly inhibited compared with control (siCtr) cells. Moreover, pre‐treatment with AG restored TGF‐β1 expression (Figure 3A,B,C) and Smad4 activation (Figure 3D) to the control levels, indicating that in metastatic PC3 cells, Glo1‐dependent MG‐H1 and AP depletion was able to drive TGF‐β1/SMAD4 signalling pathway. No differences were observed between siCtr and untreated (Ctr) cells (data not shown). We then wanted to assess whether TGF‐β1/Smad4 signalling was required for the phenotypic changes to PCa cells upon Glo1 knock‐down by the TGFβ1, inhibitor SB431542. Thus, under Glo1 knock‐down, the inhibition of TGF‐β1 signalling by SB431542^65^ potentiated Smad4 desensitization (Figure 3E), modified the expression of EMT profile‐associated molecules towards a less mesenchymal phenotype (Figure 3F) and decreased MMP‐2/MMP‐9 expression and activity (Figure 3G) and migration/invasion capabilities (Figure 3H). Comparable results were observed in DU145 cells (Figure S2). Further characterization of TGF‐β1/Smads signalling was performed by evaluating Smad2 and Smad3 phosphorylation, as well as the mRNA expression of tumour‐promoting TGF‐β1 target genes such as plasminogen activator inhibitor (PAI1),^37^, ^66^ ZEB^66^ and the high‐mobility group A2 (HMGA2),^67^, ^68^ in the same experimental conditions described above, in both DU145 and PC3 cells (Figure S3). Altogether, our results showed that Glo1‐dependent MG‐derived MG‐H1 and AP depletion triggered EMT via TGF‐β/Smad signalling pathway in metastatic PCa cell lines.

### 3.4 Identification of AP‐modified proteins

**Figure 4:**
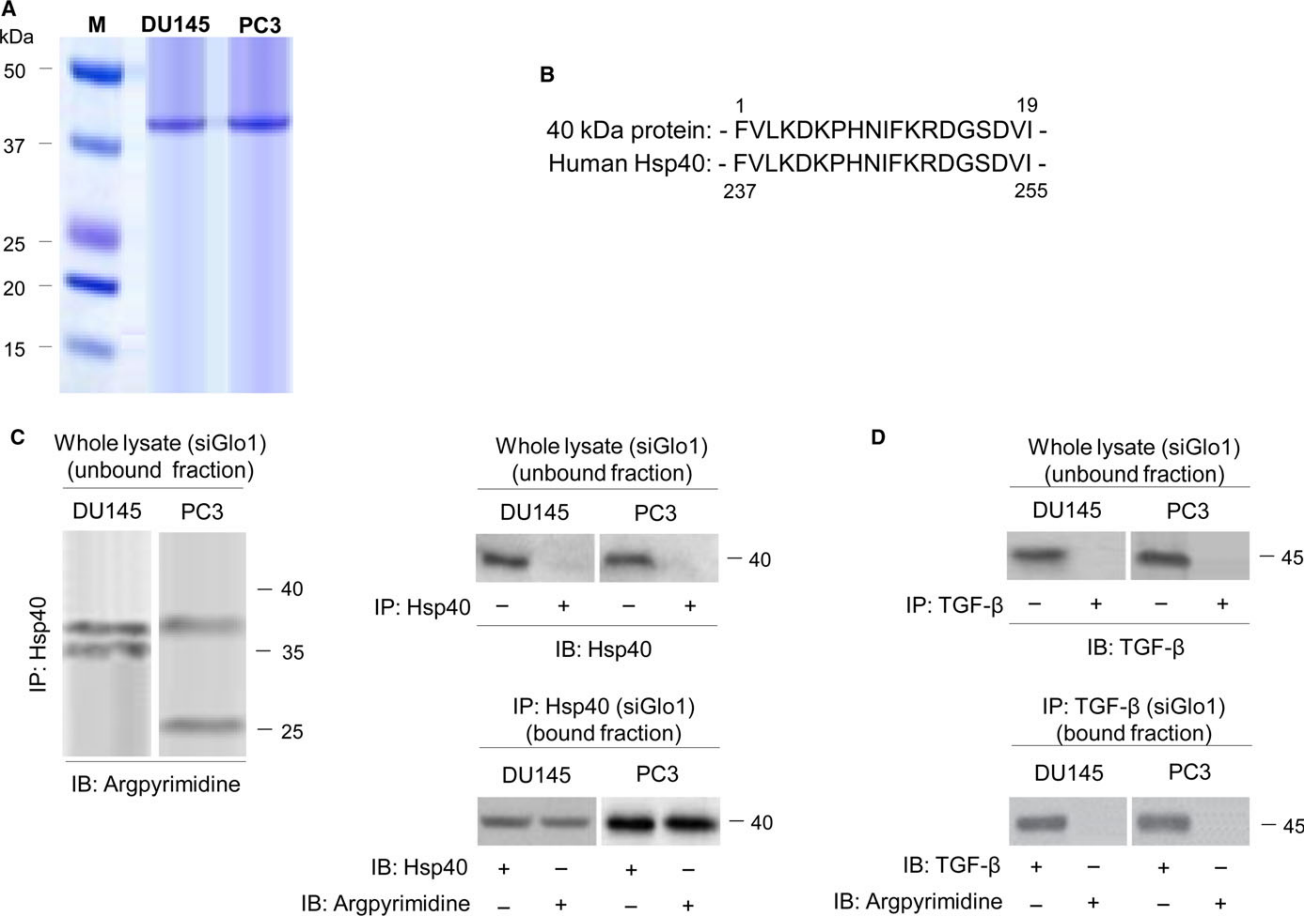
Identification of argpyrimidine (AP)‐modified proteins. (A) AP‐modified proteins were purified from the lysate of DU145 and PC3 cells upon Glo1 silencing by immunoaffinity chromatography with an anti‐Ap Ab. Chromatography fractions containing the eluted 40‐kD AP‐modified protein were identified by SDS‐PAGE and stained with Coomassie Blue. The isolated 40‐kD AP‐modified protein was then digested and resolved as individual peptides by HPLC. The internal peptides were identified as human heat‐shock protein 40 (Hsp40) upon comparison with standard sequence databases in the public domain (BLAST) (B). (C) Lysate from Glo1 silenced (siGlo1) DU145 and PC3 cells was immunoprecipitated with agarose‐coupled anti‐Hsp40 (IP: Hsp40), and unbound or bound fractions were immunoblotted (IB) with argpyrimidine or Hsp40 Abs. (D) Lysate from Glo1‐silenced (siGlo1) DU145 and PC3 cells was immunoprecipitated with agarose‐coupled anti‐TGF‐β (IP: TGF‐β), and unbound or bound fractions were IB with argpyrimidine or TGF‐β Abs. The Western blots shown are representative of three independent experiments. (−) untreated and (+) treated cells

To better understand the molecular basis of the chain of events set in motion by Glo1 depletion in PCa cells, we attempted to identify AP‐modified proteins. To this end, AP‐modified proteins were purified from lysates of DU145 and PC3 cells upon Glo1 silencing by immunoaffinity chromatography with an anti‐AP antibody. The column chromatography fraction containing the eluted 40‐kD AP‐modified protein was separated by SDS‐PAGE and visualized with Coomassie Blue (Figure 4A). To determine its identity, the purified 40‐kD AP‐modified protein was then digested and resolved as individual peptides by HPLC. As a result, the internal peptides identified as human heat‐shock protein 40 (Hsp40) upon comparison with standard sequencing databases in the public domain (BLAST [Figure 4B]), suggesting that Glo1 silencing leads to the accumulation of AP‐modified Hsp40 protein. To further confirm Hsp40 as target of MG‐mediated carbonylation, we performed immunoprecipitation (IP) experiments. Lysates from DU145 and PC3 cells upon Glo1 silencing were used for IP with agarose‐coupled anti‐Hsp40. As shown in Figure 4C, Hsp40 formed AP in both cell types, thus indicating that Hsp40 was modified by MG to form AP. As we found that TGF‐β was robustly down‐regulated in siGlo1 DU145 and PC3 cells, we wondered whether TGF‐β could also be modified by MG in these cells. IP with agarose‐coupled anti‐TGF‐β (Figure 4D) demonstrated that TGF‐β was not a target of MG‐dependent modification after IB with the AP Ab. Finally, IP with agarose‐coupled anti‐E‐cad, anti‐ZO‐1, anti‐Vim, anti‐N‐cad, anti‐Snail, anti‐MMP‐2 and anti‐MMP‐9 showed that like TGFβ1, these EMT‐associated proteins were not subject to MG modification to form AP (data not shown).

### 3.5 Loss of tumour suppressor miR‐101 induces Glo1‐dependent EMT in DU145 and PC3 cells

**Figure 5:**
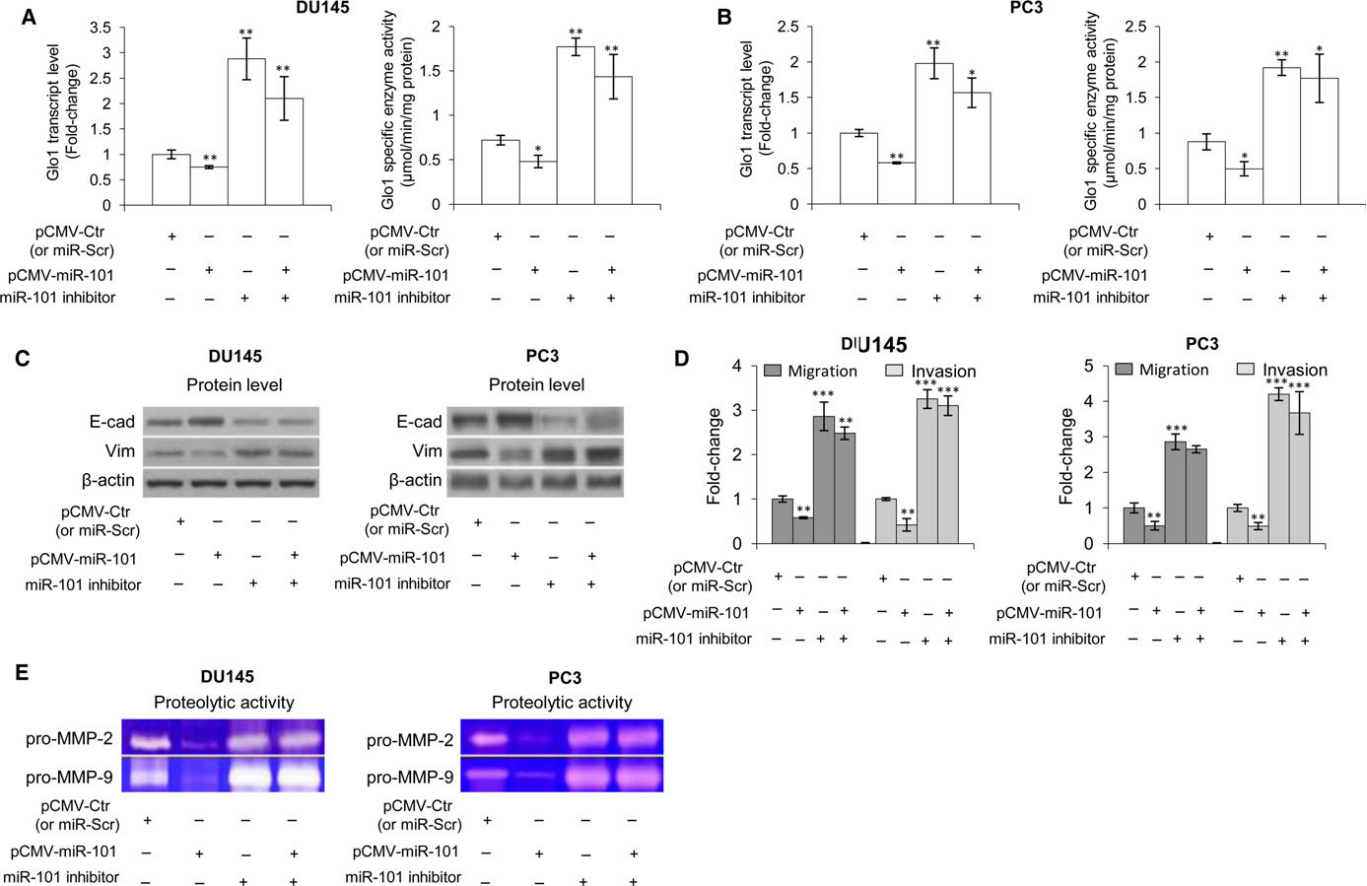
Glyoxalase 1 (Glo1) drives epithelial‐to‐mesenchymal transition (EMT), migration/invasion and MMP‐2/MMP‐9 activity under miR‐101 control in DU145 and PC3 cells. Effect of miR‐101 overexpression (pCMV‐MiR‐101) and miR‐101 inhibition on (A, B) Glo1 expression, at transcript and functional levels, evaluated by qRT‐PCR and spectrophotometric analysis, respectively, (C) EMT, evaluated by Western blot analysis of the epithelial marker E‐cadherin (E‐cad) and the mesenchymal marker vimentin (Vim), (D) migration/invasion capabilities, evaluated by specific assays, and (E) MMP‐2/MMP‐9 activity, evaluated by zymography, in both DU145 and PC3 cells. The Western blots were obtained by the appropriate Abs. The blots were stripped off the bound Abs and reprobed with anti‐β‐actin to confirm equal loading. The Western blots shown are representative of three independent experiments. The histograms indicate mean ± *SD* of three different cultures, and each was tested in triplicate. *P < .05, **P < .01, ***P < .001 vs controls (pCMV‐Ctr or miR‐Scr)

Loss of tumour suppressor microRNAs (miRNAs or miR) is an established mechanism in cancer progression. It has been reported that reduced expression or loss of miR‐101 is associated with metastasis of PCa cells.^69^, ^70^ We have recently demonstrated that miR‐101 down‐regulates Glo1 expression.^10^ Hence, we have suggested that the observed Glo1 role in sustaining the metastatic phenotype of DU145 and PC3 cells could be dependent on the reduced miR‐101 expression in these cells. After evaluating the transcript levels of miR‐101 (Figures S4A and S5A) and Glo1 (Figure S4A) in both cell lines, we demonstrated, either in DU145 or in PC3 cells, that miR‐101 depletion, achieved by a specific miR‐101 inhibitor, significantly potentiated Glo1 expression at the transcript, protein and functional levels (Figure S4B,C), EMT (Figure S4D), MMP‐2/MMP‐9 expression/activity (Figure S4E) and migration/invasion capabilities (Figure S4F), compared with miR‐Scr‐treated (control) cells. In addition, overexpression of miR‐101, achieved by transfecting cells with pCMV‐miR‐101 or miR‐control (pCMV‐Ctr), significantly down‐regulated, either in DU145 or in PC3 (Figure S6) cells, Glo1 expression, at the transcript, protein and functional levels (Figure S6A,B), reduced EMT (Figure S6C), migration/invasion capabilities (Figure S6D) or MMP‐2/MMP‐9 expression (Figure S6E) and activity (Figure S6F), compared with pCMV‐Ctr‐treated cells. More importantly, under miR‐101 overexpression, miR‐101 inhibitor restored Glo1 expression in both DU145 and PC3 cells (Figure 5A,B) as well as EMT (Figure 5C), migration/invasion capabilities (Figure 5D) and MMP‐2/MMP‐9 activity (Figure 5E), thus confirming our hypothesis. Glo1 knock‐down did not affect miR‐101 expression (Figure S5). Overall, our data suggested that loss of tumour suppressor miR‐101 induced Glo1‐dependent EMT in DU145 and PC3 cells.

### 3.6 MiR‐101 directly targets the Glo1 3′‐UTR

**Figure 6:**
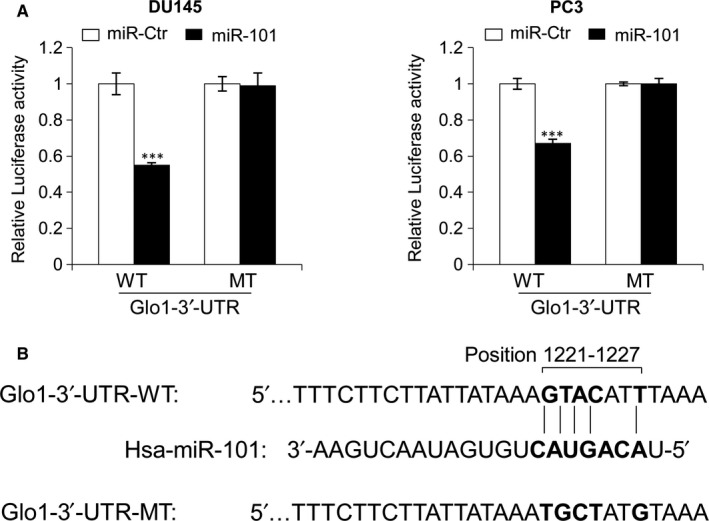
MiR‐101 directly targets the Glo1 3′‐UTR. (A) Dual luciferase reporter assay demonstrated that miR‐101 overexpression could reduce the fluorescence intensity in both DU145 and PC3 cells cotransfected with miR‐101 mimics (miR‐101), or the control (miR‐Ctr), and the wild‐type Glo1 3′‐UTR vector (WT); similar effects were not observed with the mutant‐type Glo1 3′‐UTR vector (MT). (B) Putative miR‐101‐binding sequence (bold letters) in the 3′‐UTR of Glo1 mRNA (here the DNA sequence has been shown). Mutation was generated on the Glo1 3′‐UTR sequence in the complementary site for the seed region of miR‐101, as indicated. Firefly luciferase activity of each sample was normalized by Renilla luciferase activity. The normalized luciferase activity for the miR‐Ctr‐treated cells was set as relative luciferase activity 1. Columns indicate means of at least three independent experiments conducted in duplicate; bars, SD. ***P < .001, compared with cells transfected with miR‐Ctr

To investigate whether the Glo1 3′‐UTR was a direct target of miR‐101, we constructed vectors encoding the full length of the 3′‐UTR of Glo1 mRNA to perform a dual luciferase reporter assay (Figure 6). Cotransfection experiments showed that miR‐101 significantly reduced the luciferase activity of Glo1 containing a wild‐type 3′‐UTR but not that of Glo1 containing mutant‐type 3′‐UTR (Figure 6A). These results confirmed that Glo1 is negatively regulated by miR‐101 and is a target of miR‐101. Putative miR‐101‐binding sequences of the 3′‐UTR of Glo1 mRNA are shown in Figure 6B.

### 3.7 Glo1 sustains PCa cell metastatic phenotype in cooperation with glyoxalase 2

**Figure 7:**
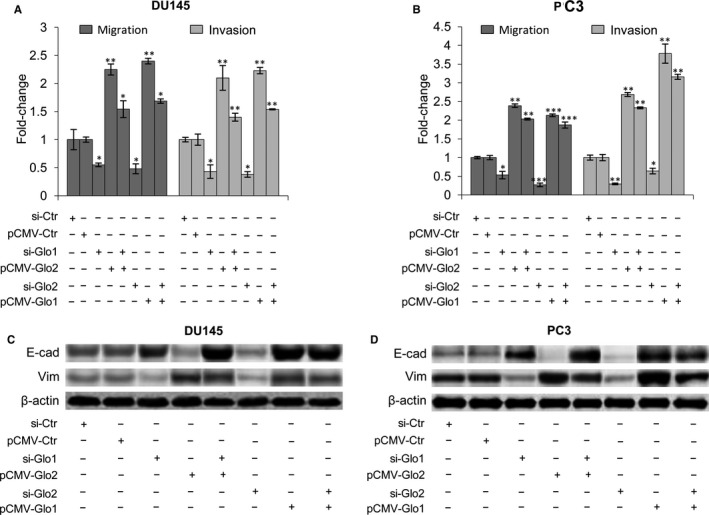
Glyoxalase 1 (Glo1) sustains prostate cancer cell metastatic phenotype in cooperation with glyoxalase 2 (Glo2). Effect of Glo1 and Glo2 ectopic expression (pCMV‐Glo1, pCMV‐Glo2) or Glo1 and Glo2 silencing (siGlo1, siGlo2) on (A, B) migration/invasion capabilities, evaluated by specific assays and (B) EMT, evaluated by Western blot analysis of the epithelial marker E‐cadherin (E‐cad) and the mesenchymal marker vimentin (Vim) in DU145 (A, C) and PC3 (B, D) cells. The Western blots were obtained by the appropriate Abs. The blots were stripped off the bound Abs and reprobed with anti‐β‐actin to confirm equal loading. The Western blots shown are representative of three independent experiments. The histograms indicate mean ± SD of three different cultures, and each was tested in triplicate. *P < .05, **P < .01, ***P < .001 vs the appropriated controls (pCMV‐Ctr or siCtr). (−) untreated and (+) treated cells

Glo1, together with Glo2, and a catalytic amount of reduced glutathione (GSH), belongs to the glyoxalase system.^71^ In this pathway, Glo1 converts MG to S‐D‐lactoylglutathione (SLG) with GSH as a cofactor, and SLG, in turn, is hydrolysed to D‐lactate by Glo2, with the regeneration of GSH. We recently demonstrated that Glo1 and Glo2 act as part of this system in aggressive PC3 cells under the control of the PTEN/PI3K/AKT/mTOR pathway and involvement of PKM2 and ERα,^45^ thus collaborating in PCa progression control. Consistent with this role, Glo1 silencing, together with Glo2 ectopic expression, and vice versa, demonstrated that both enzymes are needed to sustain DU145 and PC3 metastatic phenotype in terms of migration/invasion (Figure 7A,B) and EMT (Figure 7C,D).

### 3.8 Circulating levels of Glo1, MG‐H1, AP, miR‐101 and TGF‐β in patients with metastatic and non‐metastatic PCa

To examine whether the apparent regulatory circuit we had uncovered in PCa cell lines might be mirrored in a clinically relevant setting, we next measured circulating levels of Glo1, MG‐H1, AP, miR‐101 and TGF‐β in patients with metastatic and non‐metastatic PCa. In support of the results from the in vitro mechanistic study, we found that patients with metastatic PCa had significantly higher levels of Glo1 and TGF‐β1 and markedly lower levels of MG‐H1, AP and miR‐101 compared with patients exhibiting non‐metastatic PCa (Table 2). Moreover, a positively significant correlation was found between Glo1 and TGF‐β1 (Spearman's correlation coefficient = .84, *P* = .0012), and a negative correlation was found between Glo1 and MG‐H1 or AP or miR‐101 (Spearman's correlation coefficient = −.623 and −.640 and −.784; *P* = 0.012 and *P* = .010 and *P* = .005, respectively) in the metastatic group.

**Table 2:** Circulating levels of Glo1, AP, MG‐H1, TGF‐β1 and miR‐101 in non‐metastatic and metastatic prostate cancer (PCa) patients

	Non‐metastatic PCa (stage pT2, pT3) (n = 30)	Metastatic PCa (stage M1) (n = 30)	*P* value
Glo1 activity (mU/10^6^ RBC)			
Mean ±SD	4.73 ± 0.87	51.10 ± 4.32	<.001
MG‐H1 (μg/mL)			
Mean ± SD	7.55 ± 0.59	0.68 ± 0.27	<.001
AP (pmol/10 μmol protein)			
Mean ± SD	670.21 ± 53.48	34.87 ± 13.61	<.001
TGF‐β1 (ng/mL)			
Mean ± SD	10.05 ± 0.53	211.33 ± 8.61	<.001
miR‐101 (copies/μL serum)			
Mean ± SD	513.00 ± 43.12	17.68 ± 4.23	<.001

Stage pT2 (n = 15, cancer confined to the prostate), stage pT3 (n = 15, extraprostatic extension and/or seminal vesicle involvement). The metastatic group included patients with bone metastases (stage M1).

Glo1, glyoxalase 1; AP, argpyrimidine; MG‐H1, hydroimidazolone; TGF‐β1, transforming growth factor beta‐1; miR‐101, miRNA 101; RBC, red blood cells.

### 3.9 Metformin affects DU145 and PC3 cell metastatic phenotype, inhibits Glo1 and induces miR‐101 expression

**Figure 8:**
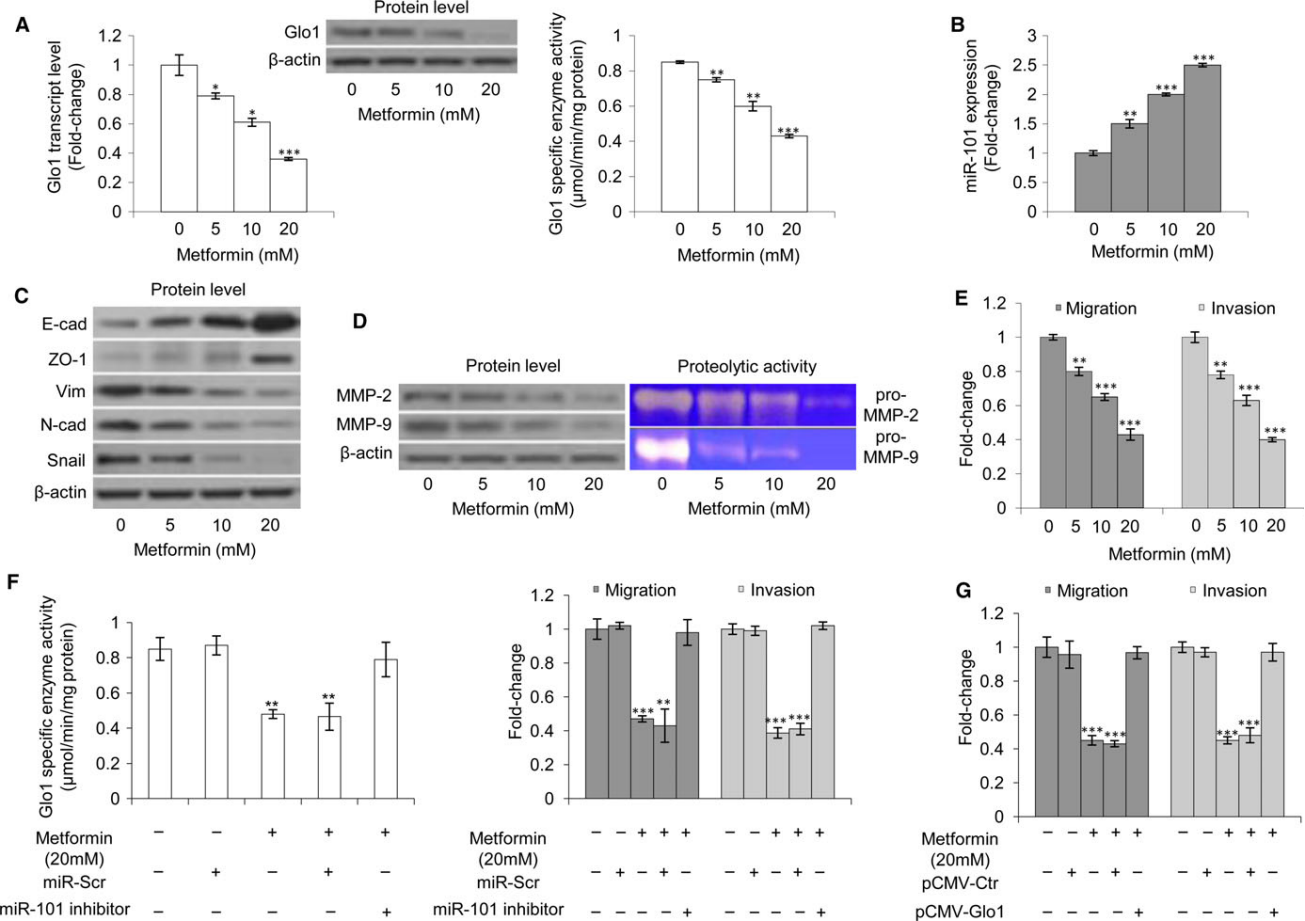
Metformin affects PC3 cell metastatic phenotype, inhibits glyoxalase 1 (Glo1) and induces miR‐101 expression. Effect of metformin on (A) Glo1 expression at transcript, protein and functional levels, evaluated by qRT‐PCR, Western blotting and spectrophotometric assay, respectively, (B) miR‐101 expression, evaluated by qRT‐PCR, (C) epithelial‐to‐mesenchymal transition (EMT), evaluated by Western blot analysis of the epithelial markers E‐cadherin (E‐cad) and zonula occludens‐1 (ZO‐1) or mesenchymal markers vimentin (Vim), N‐cadherin (N‐cad) and Snail, (D) MMP‐2 and MMP‐9 expression, evaluated by Western blotting, or activity, evaluated by zymography, and (E) migration and invasion capabilities, evaluated by specific assays. Effect of miR‐101 inhibition, under metformin administration, on (F) Glo1 enzyme activity, evaluated by spectrophotometry, and migration and invasion capabilities, evaluated by specific assays. Effect of Glo1 ectopic expression (pCMV‐Glo1), under metformin administration, on (G) migration and invasion capabilities, evaluated by specific assays. The Western blots were obtained by the appropriate Abs. The blots were stripped off the bound Abs and reprobed with anti‐β‐actin to confirm equal loading. The Western blots shown are representative of three independent experiments. The histograms indicate mean ± SD of three different cultures, and each was tested in triplicate. *P < .05, **P < .01, ***P < .001 vs untreated cells

Metformin is the most widely used anti‐diabetic drug in the world,^31^ but there is increasing evidence of its potential efficacy as an anti‐cancer drug.^31^ In particular, recent studies have suggested that metformin may have benefits especially in the field of urologic oncology.^32^, ^33^ It has been reported that Glo1 can be inhibited by metformin in endometrial cancer cells^72^, ^73^ and that miR‐101, typically lost in pancreatic cancer, can be re‐expressed following metformin treatment, and in this setting, it is associated with inhibition of cell proliferation, cell migration and invasion, and self‐renewal capacity of cancer stem cells.^74^ Therefore, we wanted to investigate whether metformin could also down‐regulate Glo1 and up‐regulate miR‐101 in our models and whether these changes might be causally linked with the rescue of the metastatic phenotype of DU145 and PC3 cells in terms of EMT, migration and invasion. In PC3 cells, we found that metformin significantly inhibited Glo1 expression (Figure 8A) and increased miR‐101 expression (Figure 8B) in a dose‐dependent manner compared with untreated control cells. Moreover, this was associated with a reversal of EMT (Figure 8C), decreased MMP‐2/MMP‐9 expression (Figure 8D), as well as decreased migratory and invasive potential (Figure 8E). More importantly, following metformin administration, miR‐101 inhibition restored Glo1 activity and migration/invasion (Figure 8F), and similarly, Glo1 overexpression did (Figure 8G). Similar results were obtained in DU145 cells (Figure S7).

## 4 DISCUSSION

The dissemination of PCa is a common, incurable aspect of the advanced disease and the primary cause of death in PCa patients. Prevention and treatment of this terminal phase of PCa require improved understanding of its molecular aetiology to obtain more insights into how to decrease morbidity and mortality in this disease. In the present study, we have, for the first time, demonstrated that Glo1 sustains the metastatic phenotype of PCa via EMT control, suggesting this metabolic protein as a potential novel molecular target in metastatic PCa control. EMT is a transdifferentiation process in which polarized epithelial cells lose their polarity and adhesion and transit to a mesenchymal cell phenotype. Consequently, EMT‐transformed cells acquire enhanced migratory capacity and elevated invasive abilities, further fostered by the activity of MMP family proteins. EMT is a crucial prerequisite for the acquisition of metastatic potential in cancer cells.^57^ However, the processes controlling EMT in malignant cells are still emerging. Here, we demonstrated that Glo1 sustains the metastatic phenotype of PCa cells via the control of EMT, thus further extending the knowledge of the factors contributing to EMT development in cancer cells. Mechanistically, we showed that Glo1 sustained EMT in DU145 and PC3 cells by suppressing the intracellular levels of MG‐H1 and AP, AGEs originating from the post‐translational modification of proteins by the Glo1 substrate, MG.^5^, ^15^ The role of AGEs in EMT is somewhat controversial. In diabetes‐associated renal diseases, AGEs can induce EMT,^59^ while in alveolar epithelial cells, AGEs can prevent EMT.^11^, ^75^ In PCa, to our knowledge, the role of AGEs in EMT had not hitherto been investigated. Our findings suggest that the specific MG‐derived AGEs, AP and MG‐H1, can play a protective role against EMT because their depletion, by Glo1, can promote cell transformation, thus supporting that part of the literature describing a similar role in other contexts.^11^, ^75^ In line with this, Glo1 would act in our PCa cell models as an oncoprotein, in agreement with others.^76^, ^77^, ^78^, ^79^ However, a study aimed at functionally identifying tumour suppressor genes in liver cancer identified and validated Glo1 as a tumour suppressor gene; knock‐down of Glo1 by shRNAs increased tumour growth in a mouse model.^80^ A similar role has been more recently described by Nokin et al.^81^ Therefore, Glo1 would appear to be a dual mediator in tumorigenesis and cancer progression, acting both as an oncogene and as a tumour suppressor, and it can be expected that different cancer types with different backgrounds and, for instance, different MG detoxification rates would react differently to MG stress. Here, we found that Hsp40 was specifically targeted by MG to form AP in both DU145 and PC3 cells. Hsps are a family of proteins that serve as molecular chaperones preventing the formation of non‐specific protein aggregates and controlling normal protein folding.^82^ Recent evidence also suggests that Hsps are actively involved in tumour cell proliferation, invasion, differentiation, metastases and cell death.^82^ In particular, Hsp40 represents a large and understudied family of cochaperones, and in recent years, the possible involvement of the Hsp40 family in tumorigenesis and malignant processes has been proposed.^82^ For example, in hepatocellular carcinoma, a member of Hsp40 family promotes tumour progression through induction of EMT,^83^ while in lung cancer cells, the expression of another member of the same family inversely correlates with invasion and metastasis, and accordingly, a direct correlation with E‐cadherin expression was observed.^84^, ^85^ In line with these latter studies, we identified a protective role for Hsp40‐modified AP with respect to EMT, as shown by the treatment with the MG scavenger, AG, which induced the rescue of metastatic phenotype under Glo1 silencing, and an inverse correlation with invasion and migration. In addition to AP‐modified Hsp40, other polypeptides that formed AP by Western blot (Figure 2B) and immunoaffinity purification (data not shown) were observed. Hence, we cannot rule out the possibility that other proteins may also play some role in the same context. Notably, our data exclude some possible candidates such as TGF‐β or the major EMT‐associated proteins, at least among those proteins MG‐modified to form AP. Whatever the case may be, we show here, for the first time, a protective role of the major MG‐derived AGE, MG‐H1, in the control of EMT.

TGF‐β is a cytokine that plays a fundamental role in various cellular functions. However, deregulation of the TGF‐β pathway can lead to various pathological conditions, including cancer. Although studies have demonstrated the tumour suppressive role of TGF‐β during the early stages of tumour development, it switches to a tumour promoter during the advanced metastatic stages of cancer.^86^, ^87^ TGF‐β is a major inducer of EMT in many neoplastic cell types. However, the molecular mechanisms by which TGF‐β induces EMT in advanced stages of cancer are poorly characterized.^57^ TGF‐β1, the most ubiquitous and best‐characterized isoform, promotes tumour progression and metastasis in advanced cancers via both Smad‐dependent pathways and Smad‐independent pathways.^57^ Smad4 is the key molecule in TGF‐β1‐induced EMT in the Smad‐dependent pathway.^64^ Here, we demonstrated that TGF‐β1/Smad4 signalling is activated by Glo1‐dependent MG‐H1 and AP depletion, thus identifying a novel mechanism, based on the Glo1/MG‐H1‐AP depletion axis, in regulating this signalling pathway in advanced PCa. We then found that MG‐H1‐AP depletion positively modulated not only TGF‐β1 expression at the message and protein levels but also its secretion. Moreover, the use of a TGF‐β1 receptor (TGF‐βR) inhibitor (SB431542) blocked TGF‐β1/Smad4 signalling, thus suggesting that MG‐H1‐AP depletion‐mediated control of TGF‐β1/Smad4 signalling may occur via an autocrine/paracrine TGF‐βR‐dependent mechanism.

MMPs are a family of endopeptidases required for extracellular matrix degradation. Among MMPs, MMP‐2 and MMP‐9 play important roles for basement membrane type IV collagen degradation during cancer progression, especially for promoting tumour migration and invasion.^88^ These two MMPs are well expressed in human PCa and PCa cell lines 89, 90 where their expression levels positively correlate with metastatic disease.^59^ In the present study, we also demonstrated, for the first time, that Glo1/MG‐H1‐AP/TGF‐β1/Smad axis controls MMP‐2 and MMP‐9 expression and activity.

**Figure 9:**
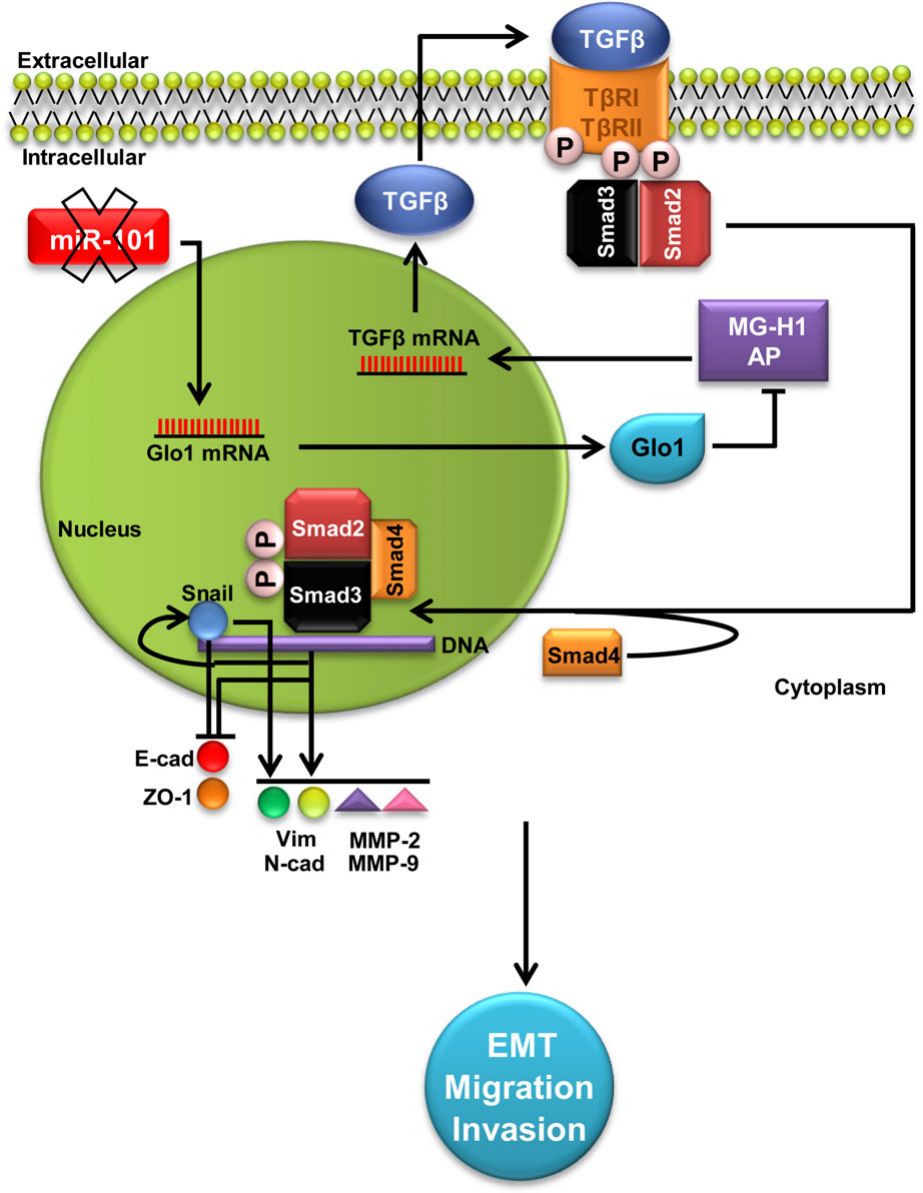
Glyoxalase 1 (Glo1) sustains the metastatic phenotype of prostate cancer cells via epithelial‐to‐mesenchymal transition (EMT) control: involvement of miR‐101, hydroimidazolone (MG‐H1), argpyrimidine (AP) and TGF‐β1/Smads signalling pathway. Glo1 up‐regulation, maintained by the decreased expression (X mark) of the tumour suppressor miR‐101, leads to MG‐H1‐AP depletion. In turn, MG‐H1‐AP depletion contributes to keep activated TGF‐β1/Smads signalling pathway that promotes EMT by inhibiting the epithelial markers E‐cadherin (E‐cad) and zonula occludens‐ (ZO‐1) and activating the mesenchymal markers vimentin (Vim) and N‐cadherin (N‐cad), together with MMP‐2 and MMP‐9, directly via Snail and/or indirectly via other TGF‐β1/Smad‐dependent EMT‐associated transcription factors. Altogether, these events sustain the invasive and migrating metastatic phenotype of prostate cancer cells

MicroRNAs (miRNAs) are small non‐coding RNAs modulating gene expression at both post‐transcriptional and post‐translational levels. Growing evidence suggests that miRNAs are important regulators of EMT^91^, ^92^ and that loss of tumour suppressor miRNA is an established mechanism in cancer progression.^69^ In particular, the tumour suppressor miR‐101 is down‐regulated in PCa and this attenuated expression is an important event in oncogenesis.^70^ Moreover, the loss or reduced expression of miR‐101 is associated with PCa cell metastasis.^69^, ^70^ Here, we demonstrated that reduced miR‐101 expression, by maintaining Glo1 up‐regulation, sustains the MG‐H1‐AP/TGF‐β1/Smad axis in metastatic DU145 and PC3 cells, suggesting a novel mechanism by which this miRNA may play a role in metastatic PCa. In agreement with Martinez‐Pacheco and colleagues who predicted and validated Glo1 as a target for miR‐101 in a different experimental setting,^93^ we too demonstrated that miR‐101 acts through imperfect base paring with the 3′‐UTR of Glo1 in our cell models. One of the major anti‐invasive effects of miR‐101 involves the histone methyltransferase enhancer of zeste homolog 2 (EZH2).^74^ Among the EMT‐associated genes evaluated in our study (E‐cad, ZO‐1, vimentin, N‐cad and Snail), only E‐cad and Snail seem to be indirectly regulated by miR‐101, via its regulation of EZH2.^94^, ^95^, ^96^, ^97^, ^98^, ^99^, ^100^ To our knowledge, this is the first demonstration of the direct negative regulation of these EMT‐associated genes by miR‐101 in PCa. Notably, computational miRNA target predictions, performed with the RNA22 version 2.0 software (http://cm.jefferson.edu/rna22),^101^ failed to predict any of the EMT identified as targets here, while the same software did identify MMP‐2 (*P* = .018), as target of miR‐101, potentially reinforcing the role of miR‐101/Glo1 axis as one of the major mechanisms in sustaining EMT, at least in DU145 and PC3 cells. The mechanistic results obtained in PC3 cells were comparable to those obtained in the other metastatic model of PCa, DU145, indicating that the described effects are likely to be applicable to PCa in general. Supporting this contention, we found that patients with metastatic PCa had significantly higher circulating levels of Glo1 and TGF‐β1 and markedly lower levels of MG‐H1, AP and miR‐101 compared with patients bearing non‐metastatic PCa. Recently, a positive correlation has been described between the circulating levels of the specific AGE, CML, and PCa progression.^20^ In particular, circulating CML levels were significantly higher in serum from high‐grade PCa patients (Gleason grade 7‐10) compared with that observed in low‐grade (Gleason grade 4‐6) PCa patients. Set against these results, circulating MG‐derived AGEs (AP and MG‐H1) levels in our PCa patient groups were lower in more aggressive than less aggressive PCa patients, suggesting that a specific pattern of AGEs may be present in the circulation of PCa patients, as it also occurs in cancer tissues from other human malignancies.^16^, ^19^ Moreover, diet‐based and/or race‐based factors might account for these specific profiles of circulating AGEs,^102^, ^103^ as has been recently demonstrated by Foster and colleagues.^20^ These investigators examined AGE levels in serum and prostate tumour specimens and identified a potential race‐specific, tumour‐dependent pattern of AGE accumulation that may be indicative of disease progression.^20^ Our data on the elevated levels of circulating TGF‐β1 in patients with metastatic PCa are in agreement with those reported in the literature.^104^, ^105^ Collectively, our results define a novel mechanism, based on miR‐101/Glo1/MG‐H1‐AP/TGF‐β/Smad axis, in the molecular aetiology of metastatic PCa (Figure 9), further extending our knowledge of the mechanisms underlying PCa metastasis and suggesting miR‐101 and Glo1 that orchestrate the mechanism, as novel potential therapeutic targets for metastatic PCa. In this regard, it is significant that we show that metformin is able to inhibit Glo1, reactivate miR‐101, and inhibit EMT, migration and invasion of metastatic PCa cells, opening new avenues of investigation for a novel potential mechanism, which up to now remain poorly explained,^74^, ^106^, ^107^ by which this drug might control metastatic phenotype in PCa. Recently, there has been debate as to how data from in vitro studies of metformin translate into in vivo activity in clinical trials, as the majority of work in vitro has been performed with concentrations varying from 1 to 100 mmol/L, predominantly between 1 and 20 mmol/L.^108^, ^109^, ^110^, ^111^, ^112^ Such concentrations would exceed levels measured in the blood of diabetic patients,^113^ although many organs such as liver, kidney or small intestine are exposed to much higher concentration of metformin compared with levels measured in the serum.^114^ Importantly, plasma membrane monoamine transporter or equilibrative nucleoside transporter ENT‐4 facilitates metformin absorption from the lumen.^115^ Other studies show the importance of the expression of the OCT family to build up intracellular levels of metformin^116^, ^117^; blocking OCT‐1, OCT‐2 and OCT‐3 inhibited OCT‐mediated transport of metformin.^118^ There is little evidence for the actual physiological concentrations of metformin achieved in normal and PCa tissues although clearly metformin could accumulate over time. Moreover, in an in vivo study that used a PCa cell xenograft model, intraperitoneal metformin treatment (1 mg/d), a dosage typically used in diabetic patients, led to a 35% reduction in tumour growth.^109^ Similarly, other studies showed that treatment of cancer cells with metformin at low concentrations (30 μmol/L) decreased their invasive capacity.^119^, ^120^

In conclusion, we demonstrate here that Glo1 sustains metastatic phenotype of DU145 and PC3 cells by controlling EMT in a novel mechanism involving the tumour suppressor miR‐101, MG‐H1‐AP and TGF‐β1/Smad signalling, thus providing valuable new insights into the pathogenesis of advanced PCa and novel options for the development of preventive and therapeutic strategies.

## CONFLICT OF INTEREST

The authors confirm that there are no conflict of interests.

## AUTHOR CONTRIBUTION

CA, RC, FR and MP performed the research; CA designed the research study; CA and VT analysed the data; CA wrote the manuscript.
