# Supplementary figures and images for: Glyoxalase 1 sustains the metastatic phenotype of prostate cancer cells via EMT control
Source: J Cell Mol Med. 2018 Mar 5;22(5):2865–83. doi: 10.1111/jcmm.13581 (PMC5908125; doi:10.1111/jcmm.13581)

Fig. S1


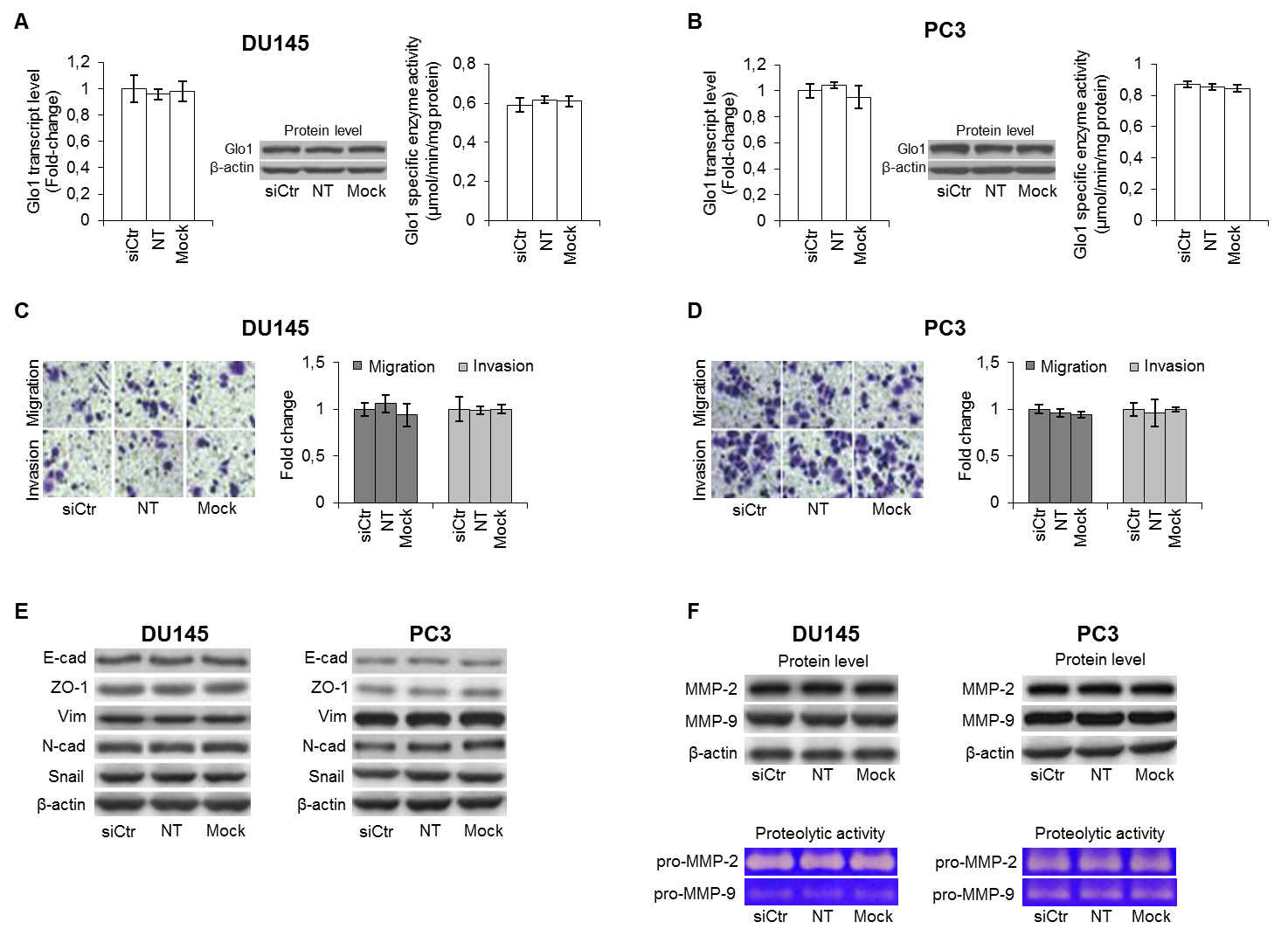


Fig. S2


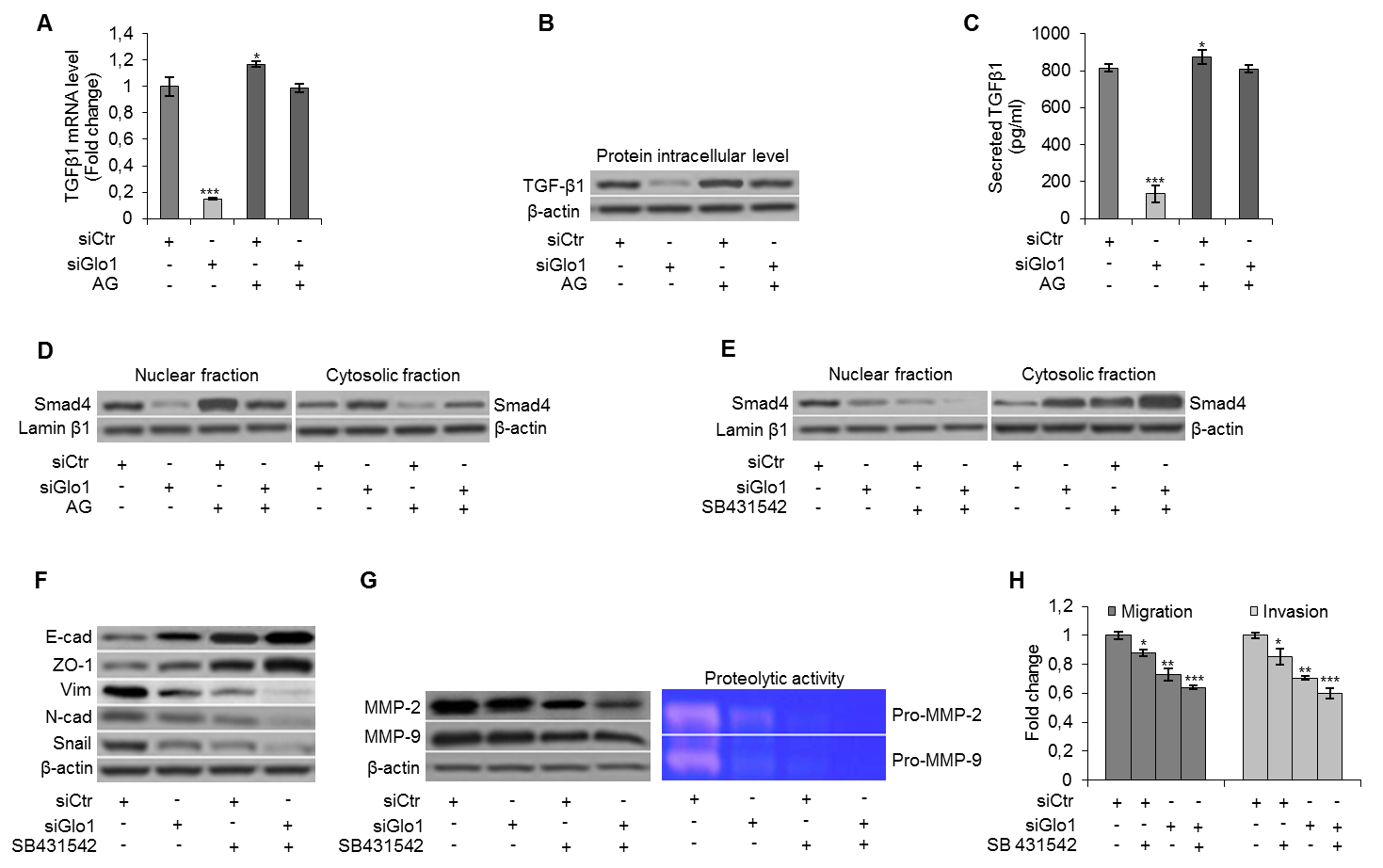


Fig. S3


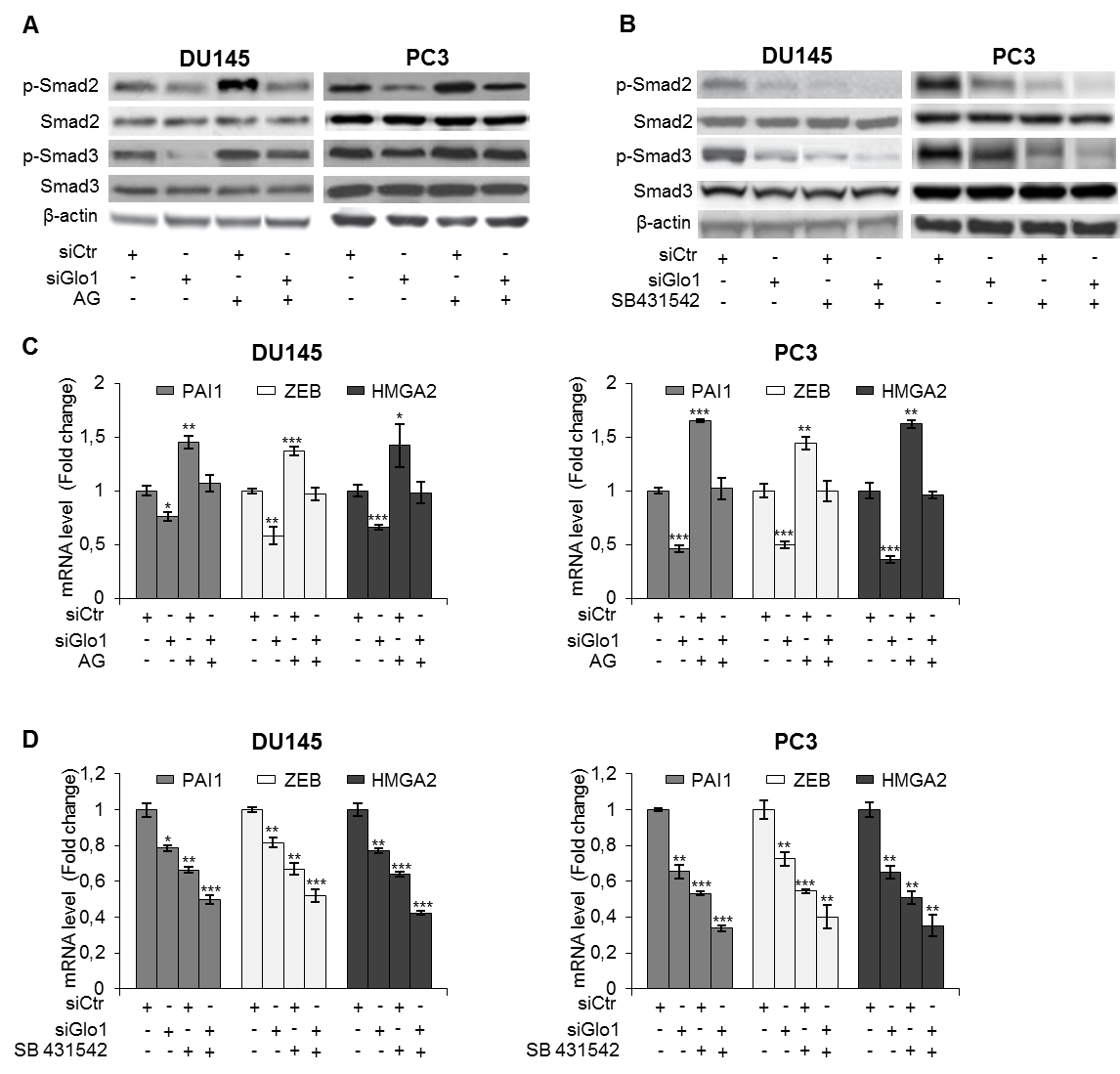


Fig. S4


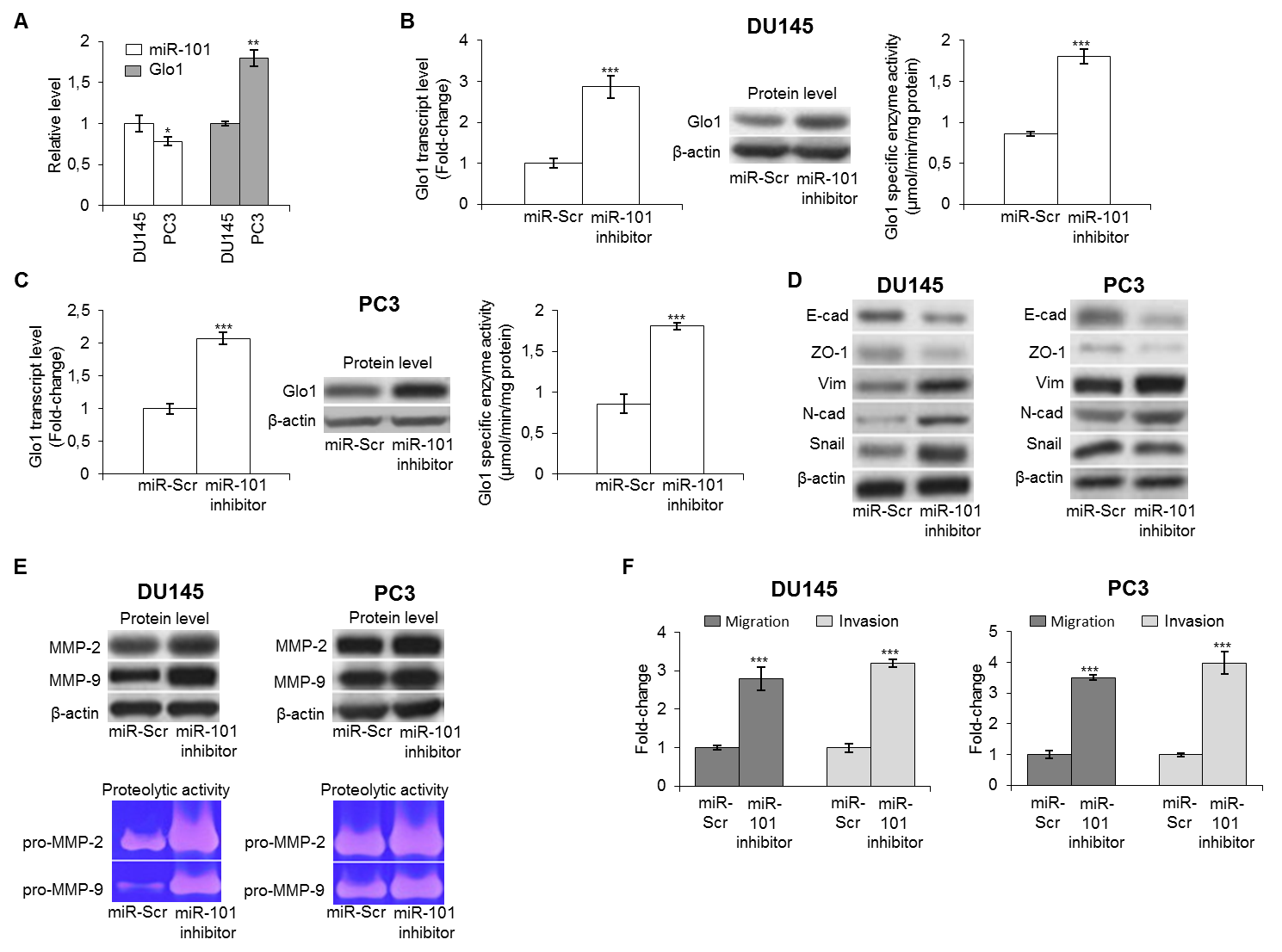


Fig. S5


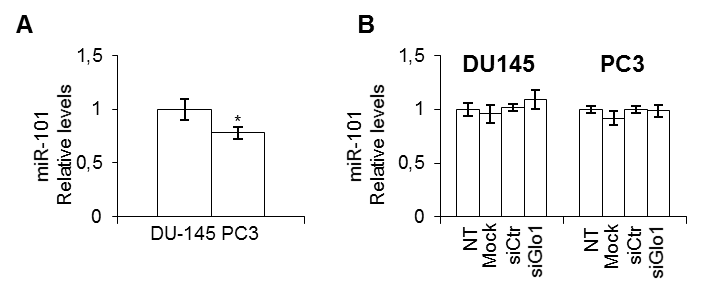


Fig. S6


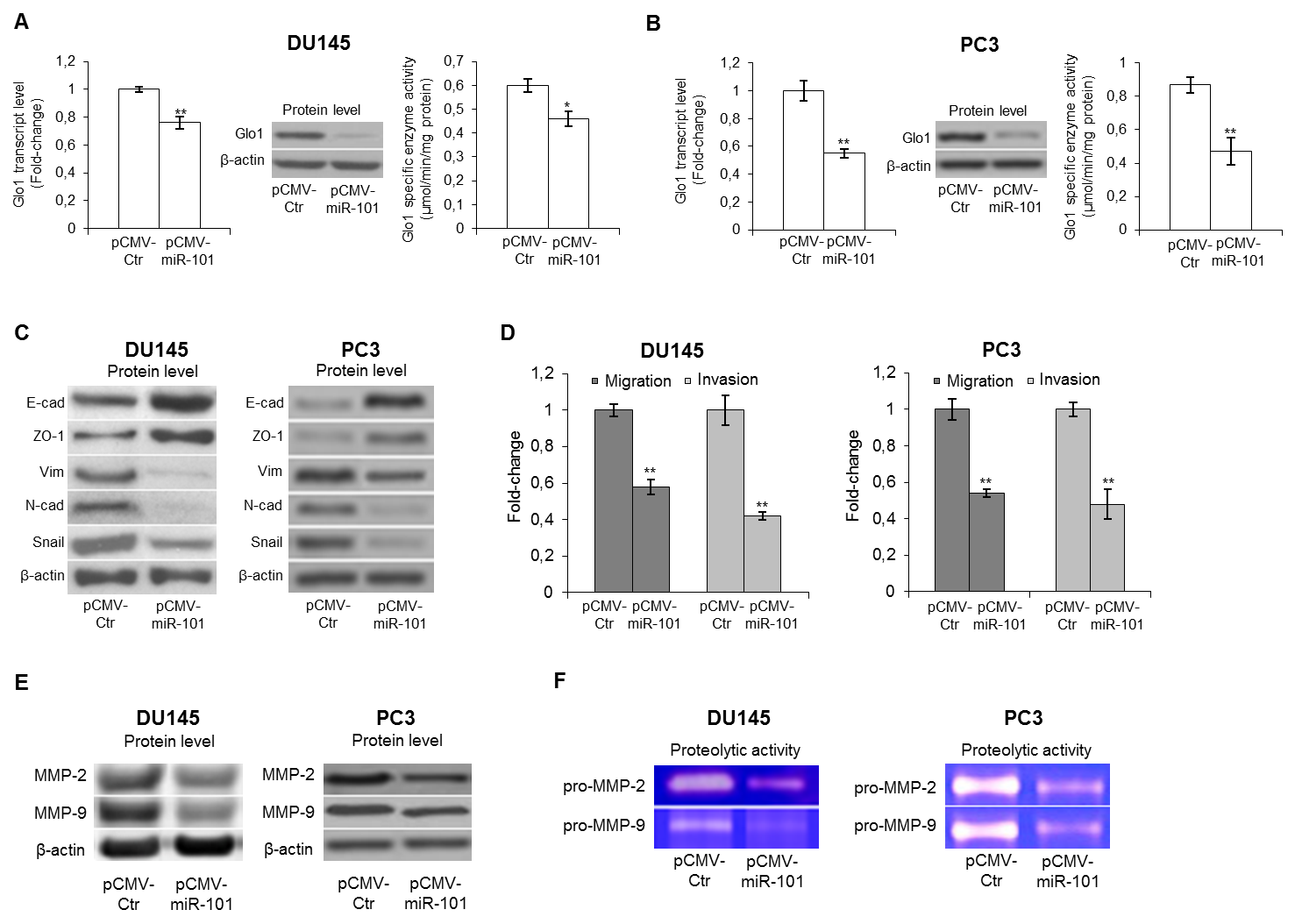


Fig. S7


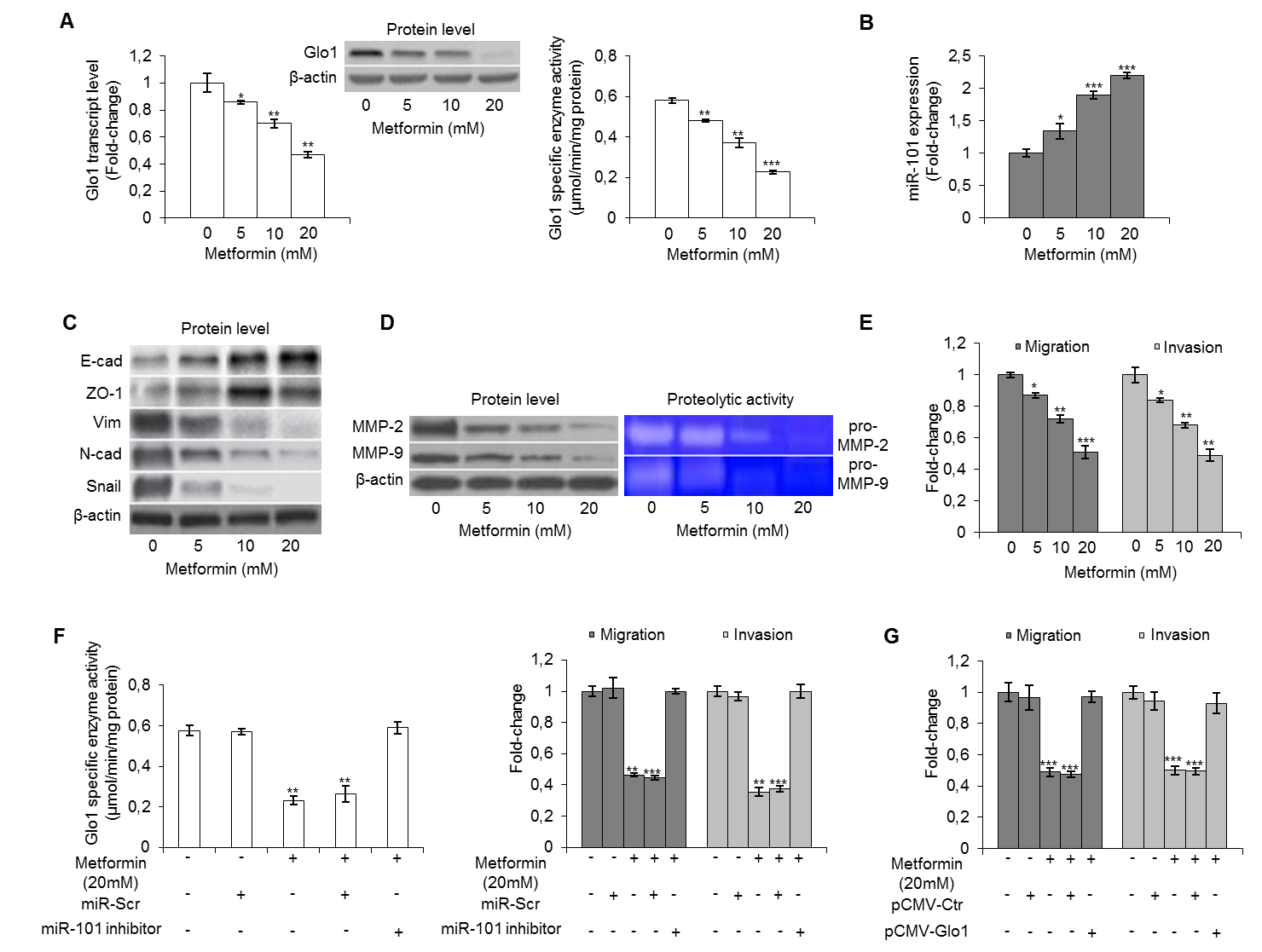

Supplement: Supplementary file 1 [file JCMM-22-2865-s001.docx]
